# The circular RNA *circBIRC6* participates in the molecular circuitry controlling human pluripotency

**DOI:** 10.1038/s41467-017-01216-w

**Published:** 2017-10-27

**Authors:** Chun-Ying Yu, Tung-Cheng Li, Yi-Ying Wu, Chan-Hsien Yeh, Wei Chiang, Ching-Yu Chuang, Hung-Chih Kuo

**Affiliations:** 1 Institute of Cellular and Organismic Biology, Academia Sinica, 11529 Taipei, Taiwan; 2 Genomics Research Center, Academia Sinica, 11529 Taipei, Taiwan; 3 Graduate Institute of Medical Genomics and Proteomics, College of Medicine, National Taiwan University, 10051 Taipei, Taiwan

## Abstract

Accumulating evidence indicates that circular RNAs (circRNAs) are abundant in the human transcriptome. However, their involvement in biological processes, including pluripotency, remains mostly undescribed. We identified a subset of circRNAs that are enriched in undifferentiated human embryonic stem cells (hESCs) and demonstrated that two, *circBIRC6* and *circCORO1C*, are functionally associated with the pluripotent state. Mechanistically, we found that *circBIRC6* is enriched in the AGO2 complex and directly interacts with microRNAs, miR-34a, and miR-145, which are known to modulate target genes that maintain pluripotency. Correspondingly, *circBIRC6* attenuates the downregulation of these target genes and suppresses hESC differentiation. We further identified hESC-enriched splicing factors (SFs) and demonstrated that *circBIRC6* biogenesis in hESCs is promoted by the SF ESRP1, whose expression is controlled by the core pluripotency-associated factors, OCT4 and NANOG. Collectively, our data suggest that circRNA serves as a microRNA “sponge” to regulate the molecular circuitry, which modulates human pluripotency and differentiation.

## Abstract (web summary)

Circular RNAs are abundant in the transcriptome and are implicated in the regulation of a range of biological processes. Here the authors identify circBIRC6 as a microRNA sponge that helps modulate human pluripotency and early lineage differentiation.

## Introduction

Circular RNAs (circRNAs) are closed RNA transcripts, generated by back-splicing of a single pre-mRNA. In this process, the 5′ terminus is covalently linked to the 3′ terminus, resulting in a scrambled exon order. The first circRNA was identified in the early 1990s^1^, however, in the subsequent two decades, only a few additional circRNAs were reported^1–3^. These circular transcripts were originally considered errors or byproducts of splicing, but with the emergence of next-generation sequencing (NGS), circRNAs are now known to be abundant and conserved among various biological systems. Moreover, the expression of circRNAs has recently been shown to be tissue specific and regulated in different biological processes, such as epithelial–mesenchymal transition (EMT) and brain development^4–6^.

The biogenesis of individual circRNAs must be tightly controlled to produce these expression profiles, and studies on circRNA generation indicate that both *cis*-elements and *trans*-acting factors are involved in regulating biogenesis. *Cis*-elements, such as canonical splicing sites, GU-AG, are necessary for circRNA biogenesis^7^, and complementary base-pair sequences in the flanking introns (e.g., Alu elements) also contribute to promoting circRNA formation by bringing two distal introns together^8, 9^. Trans-factors, such as SFs (e.g., Muscleblind and Quaking), interact with the flanking introns of circRNAs to regulate circRNA synthesis^5, 10^. Thus, it has become clear that the biogenesis of circRNAs is tightly regulated in different biological processes to allow them to fulfill their regulatory roles. However, these regulatory roles are still largely unknown. The biological functioning of circRNAs was first described by Memczak et al.^6^ and Hansen et al.^11^, who demonstrated that circRNAs can act as microRNA “sponges” to regulate gene expression. Since then, various functional roles have been reported for circRNAs in controlling biological processes^12–14^, suggesting that circRNAs may have important roles in widespread cellular functions.

Human embryonic stem cells (hESCs), which are derived from the pluripotent inner cell mass of preimplantation blastocysts, have the capacity for unlimited self-renewal and pluripotency, giving rise to many cell types in the human body^15^. A functional analysis of the core pluripotency-associated transcription factors (PATFs), NANOG, OCT4, and SOX2, has revealed that they are indispensable for the maintenance of pluripotency in hESCs^16^. In addition to transcription factors, non-coding RNAs (ncRNAs), which have little or no protein-coding potential, have also been shown to have important roles in pluripotency maintenance. For example, small ncRNAs (less than 200 nucleotides), such as the microRNAs (miRNAs), miR-302/367, and miR-372 clusters, repress the expression of differentiation-related genes in hESCs^17^. Conversely, miR-34a and miR-145 are known to repress pluripotency-associated genes to promote in vitro differentiation of hESCs^18, 19^. In addition, long ncRNAs (lncRNAs; more than 200 nucleotides), such as *lncRNA-ES1* and *lncRNA-ROR*, repress hESC differentiation by recruiting the polycomb repressive complex, PRC2, and inhibiting miRNA activity, respectively^20, 21^. Furthermore, the *trans*-spliced lncRNA, *tsRMST*, has recently been demonstrated to promote the undifferentiated status of hESCs by repressing early lineage-associated transcription factors^22^ and WNT signaling^23^ through the PRC2 complex. These studies clearly establish the importance of ncRNAs in pluripotency maintenance; however, a functional role for circRNAs in pluripotency status has not been previously reported.

To explore the functional roles of circRNAs in regulating human pluripotency, we identified and validated a subset of hESC-enriched circRNAs. Through gain-of-function and loss-of-function experiments, we further demonstrated that two circRNAs, *circBIRC6* and *circCORO1C*, are functionally associated with pluripotency maintenance and reprogramming. Furthermore, we showed that *circBIRC6* is enriched in the RNA-induced silencing complex (RISC), containing the catalytic subunit AGO2, and promotes the pluripotent state by inhibiting miR-34a-mediated and miR-145-mediated suppression of NANOG, OCT4, and SOX2 expression. Studies on *circBIRC6* biogenesis showed that the pluripotency-associated genes NANOG and OCT4 regulate expression of the SF ESRP1 (epithelial-splicing regulatory protein 1), which is responsible for the generation of *circBIRC6* in hESCs. Collectively, our results demonstrate that circRNAs participate in the molecular circuitry that controls human pluripotency.

## Results

### Identification and validation of hESC-enriched circRNAs

**Fig. 1:**
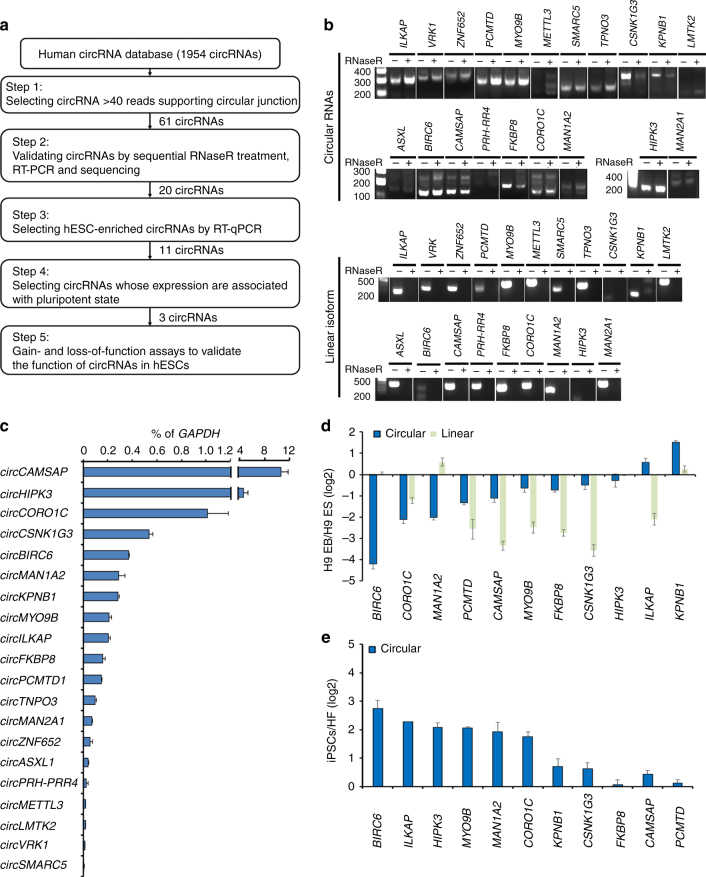
Identification and validation of hESC-enriched circRNAs. **a** Flowchart illustrating the sequence of steps for identifying and validating circRNAs enriched in hESCs. **b** RT-PCR validation of circRNAs and their linear isoforms from RNaseR-treated RNA samples isolated from hESCs. Specific primers were used to distinguish the indicated circRNAs from their linear isoforms. **c** The expression level of indicated circRNAs in hESCs by RT-qPCR analysis. The expression of circRNAs was normalized to *GAPDH*. **d** Comparison of the expression levels of indicated circRNAs and linear isoforms between hESCs and their differentiated derivatives (H9-EB) by RT-qPCR analysis. **e** Comparison of the expression levels of the indicated circRNAs between OSKM-reprogrammed iPSCs and their parental fibroblasts (HFs) by RT-qPCR analysis. Quantitative data from three independent experiments is presented as mean ± SD (error bars). *P*-values were determined by two-tailed two-sample *t*-tests (*P < 0.05; **P < 0.01; ***P < 0.001)

**Fig. 2:**
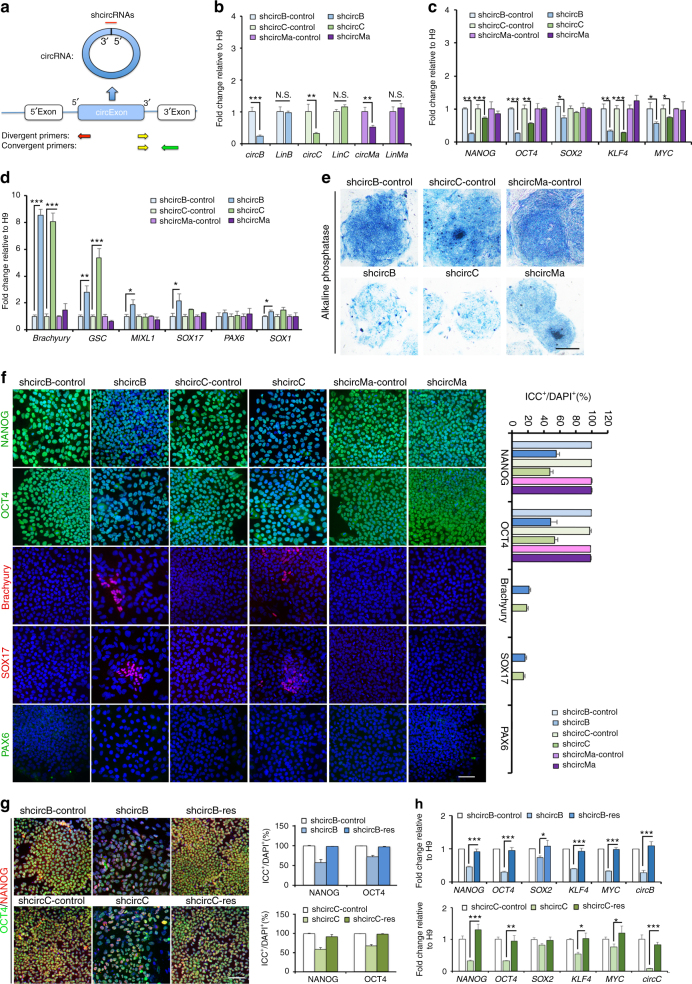
Disrupting *circBIRC6* or *circCORO1C* impairs hESC pluripotency. **a** The schematic illustrates how circular junction sites were targeted for circRNA-specific shRNAs (shcircRNAs). The schematic also shows the targeted regions for RT-qPCR primers. Divergent primers were used for circRNAs and convergent primers for the linear isoforms. **b**–**d** Total RNA isolated from hESCs transfected with shcircB, shcircC, shcircMa, or paired control virus 3 days post transduction was analyzed by RT-qPCR to determine the expression levels of **b** *circBIRC6* (circB), *circCORO1C* (circC), *circMAN1A2* (circMa), and their linear counterparts (LinB, LinC, and LinMa), and the indicated **c** pluripotency-associated genes and **d** lineage-associated genes. **e** AP staining of shcircB, shcircC, and shcircMa-transduced H9 hESCs. Scale bar: 200 μm. **f** ICC analysis of shcircB, shcircC, or shcircMa-transduced H9 hESCs 3 days post virus infection using antibodies against the pluripotency and lineage markers as indicated. The corresponding bar graphs show quantitative analysis of the percentage of cells expressing pluripotency-associated markers or lineage-associated markers. Scale bar: 20 μm. **g** ICC and quantitative comparison of NANOG and OCT4 expression between shcircB-transduced and shcircC-transduced hESCs and their counterparts rescued by overexpression of *circBIRC6* (H9-shcircB-res) or *circCORO1C* (H9-shcircC-res). Nuclei were stained with DAPI (blue). Scale bar: 20 μm. **h** RT-qPCR analysis of the expression of the indicated pluripotency-associated genes in shcircB-transduced or shcircC-transduced H9 hESCs and their rescued counterparts, H9-shcircB-res and H9-shcircC-res. Quantitative data from three independent experiments is presented as mean ± SD (error bars). *P*-values were determined by two-tailed two-sample *t*-tests (*P < 0.05; **P < 0.01; ***P < 0.001)

To identify a pool of potential circRNAs that may be expressed in hESCs, we utilized a human circRNA database that comprises candidates identified from various cell types^6^, and selected 61 strong candidates for circRNAs that had more than 40 reads supporting a circular junction (step 1 in Fig. 1a and Supplementary Data 1). Combining RT-PCR analysis that uses divergent primers to specifically target the circular junction (Fig. 2a) and sequencing validation, we detected the presence of 20 circRNAs in hESCs (step 2 in Fig. 1a). As circRNAs may share the same junction sequence as their linear *trans*-spliced counterparts, and experimental artifacts may be generated by template switching during RT-PCR^24^, we treated total RNA from hESCs with RNaseR, which digests only linear RNAs containing a free 3′ terminus and not circRNAs^25^. This further analysis confirmed the circular nature of all 20 hESC-expressed circRNAs, by showing that these molecules were resistant to RNaseR digestion, whereas the linear isoforms were not (Fig. 1b). To determine whether these circRNAs are functionally associated with pluripotency, we first examined their temporal expression pattern during embryoid body (EB) differentiation. RT-qPCR analyses showed that 11 out of the 20 hESC-expressed circRNAs were enriched in hESCs (>0.1% of *GAPDH* levels; Fig. 1c). Among the 11 hESC-enriched circRNAs, nine were downregulated in EBs (Fig. 1d). In addition, all hESC-enriched circRNAs were upregulated in induced pluripotent stem cells (iPSCs) as compared with the parental fibroblasts (Fig. 1e). Notably, the RT-qPCR analyses also revealed that circRNA expression was often co-regulated with the corresponding linear isoform during differentiation. This was true for all circRNAs except for *circBIRC6*, *circMAN1A2*, and *circILKAP* (Fig. 1d), suggesting that these three circRNAs and their linear counterparts are differentially regulated with cell status. We further confirmed the presence of transcripts for *circBIRC6*, *circCORO1C*, and *circMAN1A2*, which exhibited the highest association with pluripotency status (fold change > 4 during differentiation and reprogramming), in hESCs by Northern blot analysis using probes that specifically target the circular junction of these circRNAs (Supplementary Fig. 1). This analysis conclusively excluded that these circRNAs were in vitro artifacts resulting from template switching. Collectively, we identify a set of circRNAs that are enriched in hESCs and iPSCs, and show that their expression is associated with the pluripotent state.

### Disrupting *circBIRC6* or *circCORO1C* impairs hESC pluripotency

**Fig. 3:**
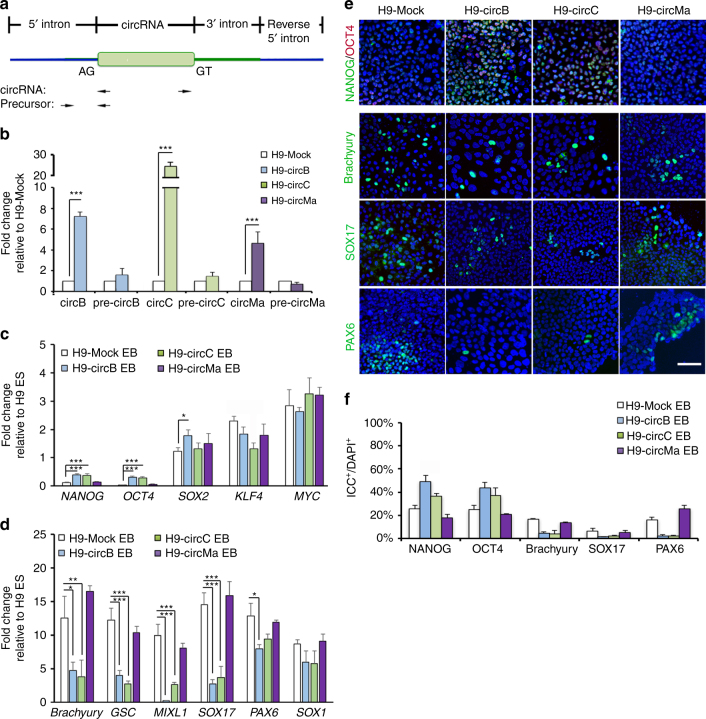
*C* *ircBIRC6* or *circCORO1C* promote hESC pluripotency**. a** Schematic illustrating the major components of circRNA minigene constructs. Arrows indicate the placement and direction of RT-qPCR primers used to amplify circRNA or precursor transcripts. **b**–**d** RT-qPCR analysis of the expression of **b** circRNAs and their precursor transcripts, and the indicated **c** pluripotency-associated genes and **d** lineage-associated genes in hESCs transfected with a circRNA minigene (H9-circB, H9-circC, and H9-circMa) cultured under differentiation conditions. **e** ICC analysis and **f** quantitative analysis of the expression of the pluripotency and lineage markers as indicated in hESCs transfected with a circRNA minigene (H9-circB, H9-circC, and H9-circMa) and cultured under differentiation conditions. Nuclei were stained with DAPI (blue). Scale bar: 20 μm. Quantitative data from three independent experiments is presented as mean ± SD (error bars). *P*-values were determined by two-tailed two-sample *t*-tests (*P < 0.05; **P < 0.01; ***P < 0.001)

To determine whether the hESC-enriched circRNAs function in pluripotency maintenance, we disrupted *circBIRC6*, *circCORO1C*, or *circMAN1A2* expression in hESCs by overexpressing small hairpin RNAs (shRNAs) that specifically target the circular junction of each circRNA (shcircRNAs in Fig. 2a). Notably, expressing shcircRNAs in hESCs only disrupted the expression of circRNAs and not their linear counterparts, as demonstrated by RT-qPCR (Fig. 2b). After expressing shcircRNA in hESCs, we found that alkaline phosphatase (AP) activity was significantly reduced in *circBIRC6-*knockdown (H9-shcircB) and *circCORO1C*-knockdown (H9-shcircC), but not in *circMAN1A2-*knockdown (H9-shcircMa) hESCs, as compared with hESCs that express paired control shRNAs (Fig. 2e). RT-qPCR further revealed that expression levels of the PATFs, *NANOG*, *OCT4*, *KLF4*, and *MYC*, were downregulated in H9-shcircB and H9-shcircC (Fig. 2c), whereas expression of the lineage-related transcription factors (LRTFs) *Brachyury*, *GSC*, *MIXL1*, *SOX17*, and *SOX1* was significantly upregulated (Fig. 2d). Immunocytochemistry (ICC) showed that the number of NANOG^+^ and OCT4^+^ cells was significantly reduced in H9-shcircB and H9-shcircC, whereas the number of Brachyury^+^ and SOX17^+^ cells was significantly increased (Fig. 2f). To exclude the possibility of off-target effects from the shcircRNA constructs, we re-expressed *circBIRC6* and *circCORO1C* (Fig. 3a for vector construction) in H9-shcircB and H9-shcircC, respectively. After confirming, the expression levels of *circBIRC6* and *circCORO1C* were rescued in H9-shcircB-res and H9-shcircC-res cells by RT-qPCR analysis (Fig. 2h), we performed RT-qPCR and ICC to show that the expression levels of PATFs and the number of NANOG^+^ or OCT4^+^ cells were similarly rescued (Fig. 2g, h). Consistent with our hESC results, various assays showed that disruption of *circBIRC6* and *circCORO1C* expression downregulated PATFs expression in iPSCs (Supplementary Fig. 2).

To rule out the possibility that the above observations are caused by linear *BIRC6*, we transduced hESC with shRNAs that specifically targets linear *BIRC6* (shLinB), or the common exon of both *circBIRC6* and linear *BIRC6* (shcircB-2). After confirming the specific disruption of shLinB and shcircB-2 on their target isoforms by RT-qPCR (Supplementary Fig. 3a), we found that the AP activity was decreased in shcircB-2, but not shLinB, transduced hESCs (Supplementary Fig 3e). RT-qPCR and ICC further showed the downregulation of PATFs (*NANOG*, *OCT4*, *KLF4*, and *MYC*), and miRNA-targeting gene expression (*MDM4*, *SIRT1*, *ADAM17*, and *NEDD9*), and upregulation of LRTFs expression (*Brachyury*, *GSC*, *MIXL1*, and *SOX17*) in shcircB-2-transduced hESCs, as compared to shLinB-transduced hESCs (Supplementary Fig. 3b–d). Further, ICC analyses revealed a decreased number of NANOG^+^ and OCT4^+^ cells, and an increased number of Brachyury^+^ and SOX17^+^ cells in shcircB-2-transduced hESCs (Supplementary Fig. 3f), suggesting that *circBIRC6*, but not linear *BIRC6*, is critical for pluripotency maintenance. Altogether, these results indicate that *circBIRC6* and *circCORO1C* may be functionally involved in the maintenance of hESC pluripotency.

### Expressing *circBIRC6* or *circCORO1C* retains hESC pluripotency

To examine whether the aforementioned circRNAs maintain pluripotency, we performed gain-of-function experiments by overexpressing circRNAs with minigene constructs and examined the expression of circRNAs and their precursors by RT-qPCR and Northern analyses, using primer pairs that specifically target the circular junction site and the 5′ intron–exon junction (Fig. 3a, see details in “Methods”). The result showed that expression levels of *circBIRC6*, *circCORO1C*, and *circMAN1A2* were all significantly increased in hESCs transfected with minigenes expressing *circBIRC6* (H9-circB), *circCORO1C* (H9-circC), or *circMAN1A2* (H9-circMa) compared with mock-transfected controls, whereas there was no increase in un-spliced precursors (Fig. 3b; Supplementary Fig. 4a). RT-qPCR and ICC staining revealed that ectopic expression of these circRNAs in hESCs, cultured under ESC culture conditions, did not reduce the number of cells expressing PATFs or increase the number of cells expressing LRTFs, suggesting that high expression levels of circRNAs in hESCs did not cause a loss of pluripotency (Supplementary Fig. 4b, c). However, ectopic expression of *circBIRC6* or *circCORO1C*, but not *circMAN1A2*, in differentiating hESCs upregulated expression of the PATFs, *OCT4*, *NANOG*, and *SOX2*, and downregulated expression of the LRTFs *Brachyury*, *GSC*, *MIXL1*, *SOX17*, and *PAX6* (Fig. 3c, d). Accordingly, ICC analysis revealed an increased number of NANOG^+^ and OCT4^+^ cells in differentiated H9-circB and H9-circC, and a decreased number of Brachyury^+^, SOX17^+^, and PAX6^+^ cells (Fig. 3e, f). These results show that *circBIRC6* and *circCORO1C*, but not *circMAN1A2*, maintain pluripotency marker expression in differentiated hESCs.

### Expressing *circBIRC6* or *circCORO1C* promotes reprogramming

**Fig. 4:**
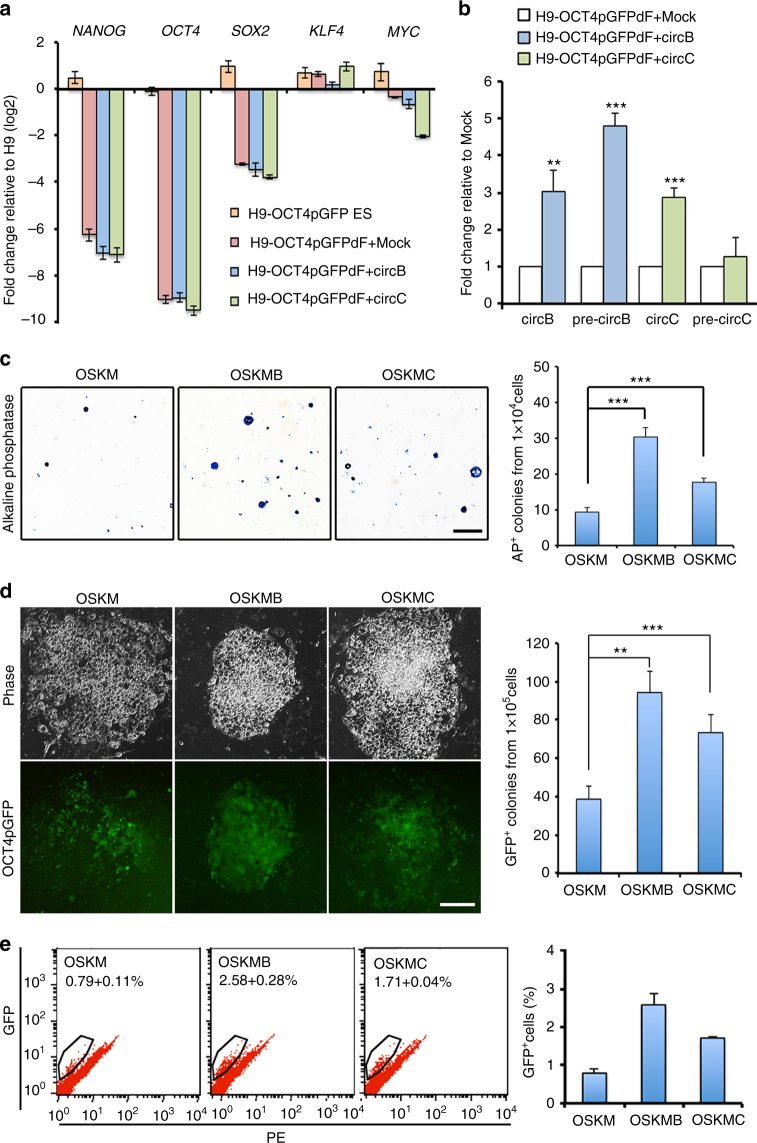
*CircBIRC6* or *circCORO1C* promotes reprogramming. **a** RT-qPCR analysis of the expression of the indicated pluripotency-associated genes in H9-OCT4pGFP, H9-OCT4pGFPdF, and H9-OCT4pGFPdF cells transduced with a *circBIRC6* (H9-OCT4pGFPdF+circB) or *circCORO1C* (H9-OCT4pGFPdF+circC) minigene. **b** RT-qPCR analysis of the expression of circRNA and its precursor transcripts in H9-OCT4pGFPdF+circB and H9-OCT4pGFPdF+circC cells using primers specific for the indicated circRNAs and circRNA precursors (pre-circB and pre-circC). **c** AP staining and quantitative analysis of AP^+^ colonies among H9-OCT4pGFPdF cells transfected with OSKM or co-transfected with OSKM and circRNA minigenes (OSKMB and OSKMC). Scale bar: 400 μm. **d** Reactivation of OCT4pGFP in H9-OCT4pGFPdF cells and quantitative analysis of OCT4pGFP^+^ colonies of H9-OCT4pGFPdF cells transfected with OSKM or co-transfected with OSKM and circRNA minigenes (OSKMB and OSKMC) 21 days post transduction. Scale bar: 50 μm. **e** Quantitative FACS analysis of OCT4pGFP^+^ cells in OSKM, OSKMB, and OSKMC 28 days post reprogramming. Quantitative data from three independent experiments is presented as mean ± SD (error bars). *P*-values were determined by two-tailed two-sample *t*-tests (*P < 0.05; **P < 0.01; ***P < 0.001)

As our results demonstrated that *circBIRC6* and *circCORO1C* contribute to human pluripotency maintenance, it was of interest to know whether their expression is sufficient to reprogram somatic cells into iPSCs. To this end, we first generated pluripotency reporter hESCs by knocking green fluorescent protein (GFP) into the endogenous OCT4 promoter using a CRISPR and Cre-loxP system (H9-OCT4pGFP; Supplementary Fig. 5a, b). Next, we differentiated H9-OCT4pGFP hESCs into a homogeneous population of fibroblast-like cells (H9-OCT4pGFPdFs) that do not express the OCT4pGFP transgene or PATFs (Fig. 4a; Supplementary Fig. 5b). To determine whether circRNAs are sufficient to promote reprogramming, we then expressed *circBIRC6* or *circCORO1C* minigene constructs in H9-OCT4pGFPdFs. The RT-qPCR analyses showed that *circBIRC6* and *circCORO1C* expression were significantly increased in H9-OCT4pGFPdFs transfected with the corresponding minigene construct (Fig. 4b). The expression of *circBIRC6* or *circCORO1C* alone did not reactivate the PATFs *OCT4*, *SOX2*, and *NANOG* in H9-OCT4pGFPdFs (Fig. 4a), suggesting that expression of *circBIRC6* or *circCORO1C* alone is insufficient to restore pluripotency status in fibroblasts. To determine whether *circBIRC6* or *circCORO1C* may improve the efficiency of TF-mediated reprogramming, we infected H9-OCT4pGFPdF with Sendai virus encoding OCT4, SOX2, KLF4, and c-MYC (OSKM), with or without co-expression of circRNA. On day 14 after viral infection, RT-qPCR analysis showed that the expression levels of endogenous *NANOG*, *OCT4*, and *SOX2* were increased in H9-OCT4pGFPdF populations co-expressing OSKM and *circBIRC6* (OSKMB) or *circCORO1C* (OSKMC) compared with H9-OCT4pGFPdFs expressing only OSKM (Supplementary Fig. 5c). By day 21 after viral infection, small compact colonies started to appear among fibroblasts undergoing reprogramming. Further AP staining and quantitative analyses showed that the numbers of AP^+^ and OCT4-GFP^+^ colonies were significantly increased in OSKMB or OSKMC compared with OSKM cells (Fig. 4c, d). By day 28 post infection, fluorescence-activated cell sorting (FACS) showed that the number of reprogrammed OCT4-GFP^+^ iPSCs was also significantly increased in OSKMB or OSKMC compared OSKM cells (Fig. 4e). These results suggest that *circBIRC6* and *circCORO1C* potentiate OSKM-mediated reprogramming.

### *CircBIRC6* acts as a sponge for miR-34a and miR-145 in hESCs

**Fig. 5:**
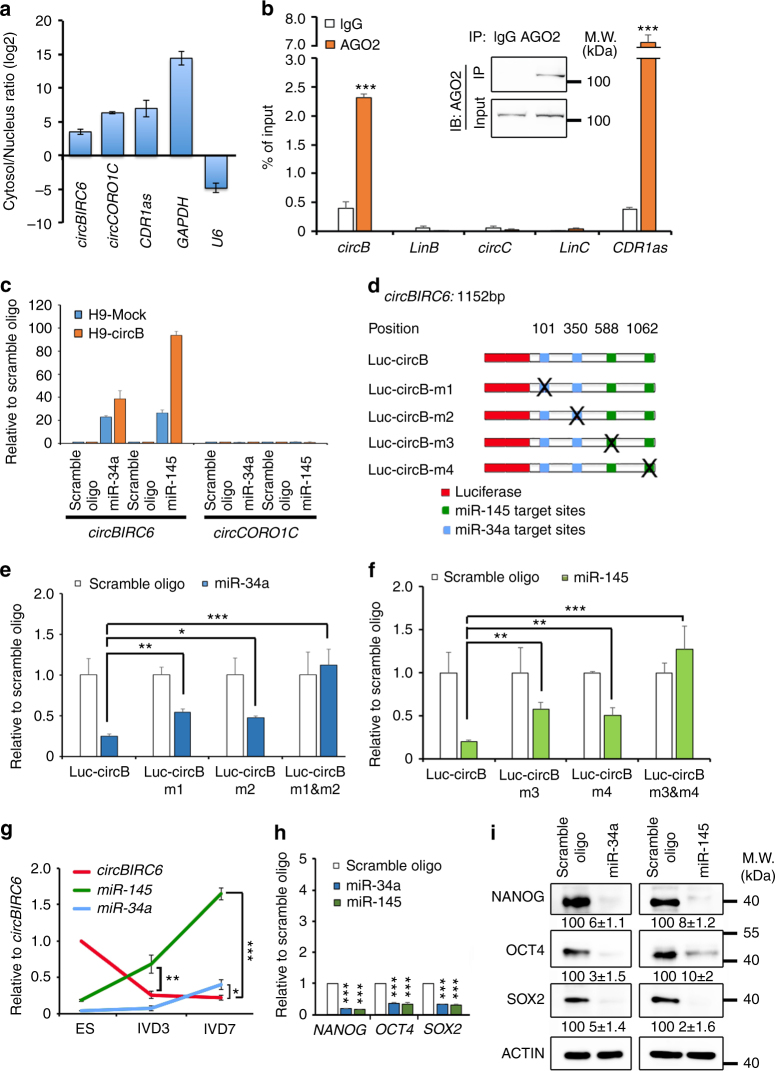
*C* *ircBIRC6* interacts with and sequester miR-34a and miR-145. **a** RT-qPCR analysis of cytoplasmic-to-nuclear expression ratios of *circBIRC6*, *circCORO1C*, *CDR1as*, *GAPDH*, and *U6* in hESCs. **b** RIP analysis of *circBIRC6*, linear *BIRC6 (linB)*, *circCORO1C*, linear *CORO1C (LinC)*, and *CDR1as* in hESCs using antibodies against AGO2. Immunoblotting (IB) of immunoprecipitated (IP) AGO2 protein is shown. The RIP enrichment of the AGO2-associated circular RNAs (as indicated) was measured by RT-qPCR, and each value was normalized to the level of input RNA used in RIP analysis. **c** RT-qPCR analysis of *circBIRC6* or *circCORO1C* pulled-down by biotinylated miR-34a or miR-145 mimics in hESCs. **d** Schematic illustration showing the 3′ UTR of luciferase reporters containing the complete *circBIRC6* sequence (luc-circB) or *circBIRC6* sequence with deletions of miR-34a (luc-circBm1 and luc-circBm2) or miR-145 (luc-circBm3 and luc-circBm4) binding sites. **e**, **f** Reporter assays showing the luciferase activity of luc-circB and luc-circBm1-m4 in 293T cells co-transfected with **e** miR-34a or **f** miR-145 mimics, or a scrambled oligonucleotide (control). **g** RT-qPCR analysis of *circBIRC6*, *miR-34a*, and *miR-145* expression during hESC differentiation. IVD: in vitro differentiation day. **h** RT-qPCR and **i** immunoblot analyses of the indicated gene and protein expression in hESCs transfected with miR-34a or miR-145 mimics, or scrambled oligonucleotide 3 days post transfection. Protein level quantification was normalized to actin and shown below each panel. Quantitative data from three independent experiments is presented as mean ± SD (error bars). *P*-values were determined by two-tailed two-sample *t*-tests (*P < 0.05; **P < 0.01; ***P < 0.001)

It is known that circRNAs can act as miRNA sponges to regulate gene expression in different cell types^6, 13, 26^. To determine whether *circ* *BIRC6* and *circ* *CORO1C* regulate pluripotency through the same mechanism, we first examined the relative expression levels of circRNAs in the cytoplasmic and nuclear compartments of hESCs. RT-qPCR results demonstrated that *circ* *BIRC6* and *circ* *CORO1C* were enriched in the cytoplasm (Fig. 5a). RNA immunoprecipitation (RIP) assays further showed that *circBIRC6*, but not *circCORO1C* or either linear isoform, was enriched in AGO2 immunoprecipitates (Fig. 5b), suggesting that only *circBIRC6* possesses miRNA-related functions. To determine which miRNAs potentially interact with *circBIRC6*, we analyzed putative miRNA-targeting sites on the *circBIRC6* sequence using a probability of interaction by target accessibility (PITA) algorithm^27^. Selecting miRNA-targeting sites with strong binding energy (Δ*G* < −14 kcal mol^−1^) and allowing one G:U wobble pair or one mismatch in the miRNA seed regions, we found 431 sites that were targeted by 306 different miRNAs (Supplementary Data 2). Among the 95 miRNAs having multiple targeting sites on *circBIRC6*, miR-34a, and miR-145 have been reported to induce hESC differentiation by directly suppressing the expression of pluripotency-associated genes^18, 19^. Thus, we asked whether *circBIRC6* was capable of regulating pluripotency in hESCs through interactions with miR-34a and/or miR-145. To this end, we transfected 3′ terminal-biotinylated miR-34a and miR-145 mimics into H9-circB, and precipitated RNA transcripts that interact with these mimics by streptavidin beads. RT-qPCR analyses revealed that *circBIRC6* was enriched in the precipitates of miR-34a and miR-145 mimics compared with precipitates of scrambled oligonucleotide (Fig. 5c). Next, we carried out luciferase reporter assays and showed that miR-34a or miR-145 expression significantly reduced the luciferase activity of a reporter containing the complete *circBIRC6* sequence appended to the 3′-untranslated region of luciferase (luc-circB), whereas the luciferase activity of luc-circB containing mutated miR-34a and miR-145 binding sites (luc-circBm1-m4) was not noticeably affected (Fig. 5d–f). For circRNAs to act as miRNA sponges and regulate miRNA activity, the relative expression level of circRNAs and their targeted miRNAs should be within an appropriate range. Accordingly, Northern blotting using probes specific for *circBIRC6* showed the downregulation of *circBIRC6* expression in differentiated hESCs (Supplementary Fig. 6) and RT-qPCR showed that miR-34a and miR-145 expression levels were lower than that of *circBIRC6* in undifferentiated hESCs, but were higher than those of *circBIRC6* in differentiated hESC derivatives (Fig. 5g). Taken together, these results suggest that *circBIRC6* may exert its function through direct interactions with miR-34a and miR-145.

**Fig. 6:**
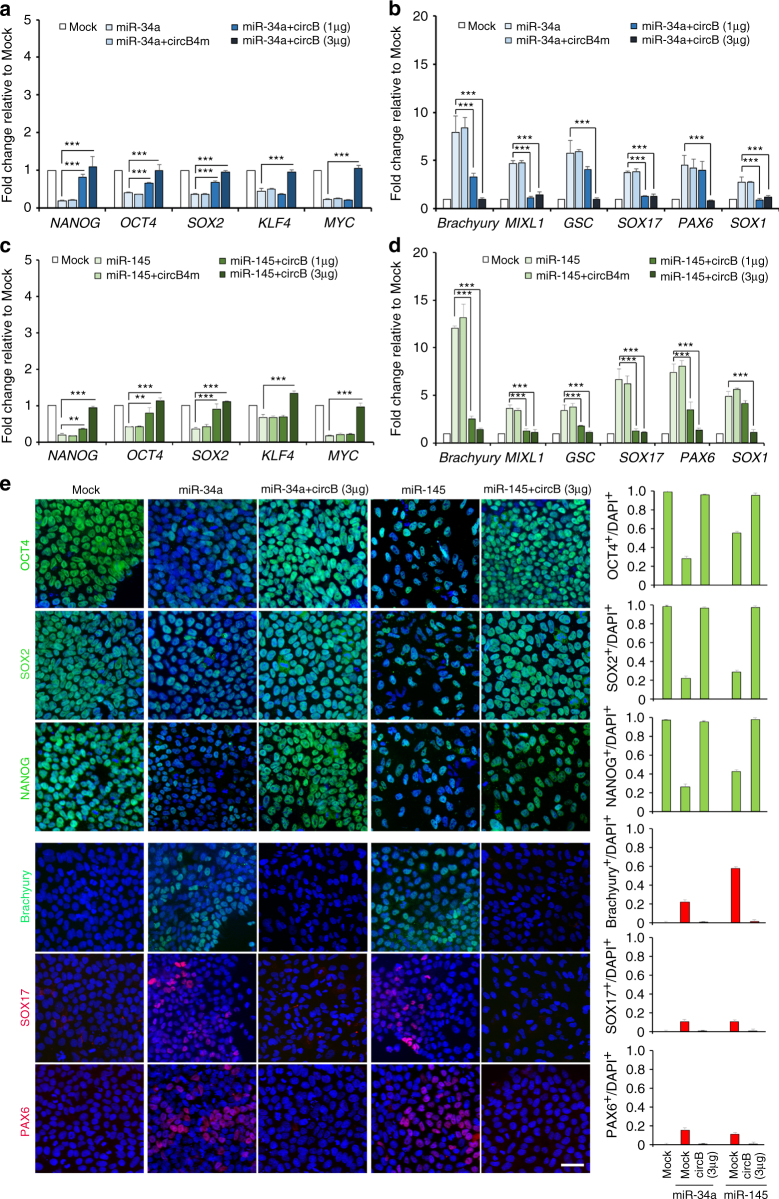
*C* *ircBIRC6* serves as a sponge for miR-34a and miR-145 in hESC. **a**, **b** RT-qPCR analysis of the expression of the indicated **a** pluripotency-associated genes and **b** lineage-associated genes in hESCs transfected with miR-34a mimics, or co-transfected with *circBIRC6* minigene construct (miR-34a+circB (1 μg or 3 μg)) or *circBIRC6* minigenes harboring deleted miR-34a and miR-145 binding sites (circB4m). **c**, **d** RT-qPCR analysis of the expression of the indicated **c** pluripotency-associated genes and **d** early lineage-associated genes in hESCs transfected with miR-145 mimics, or co-transfected with *circBIRC6* minigene construct (miR-145+circB (1 μg or 3 μg)) or co-transfected with circB4m. **e** ICC analysis of the indicated pluripotency-associated markers and lineage-associated markers in hESCs transfected with miR-34a or miR-145 mimics, or co-transfected with a *circBIRC6* minigene construct (miR-34a+circB or miR-145+circB). Nuclei were stained with DAPI. Scale bar: 20 μm. A quantitative analysis of cells expressing the indicated pluripotency-associated or early lineage-associated markers is shown in the right-hand panel. Quantitative data from three independent experiments is presented as mean ± SD (error bars). *P*-values were determined by two-tailed two-sample *t*-tests (*P < 0.05; **P < 0.01; ***P < 0.001)

To ascertain whether *circBIRC6* contributes to pluripotency maintenance by acting as a sponge for miR-34a and miR-145, we transfected hESCs with miR-34a or miR-145 mimics, and examined their impact on hESC status. RT-qPCR results showed that overexpression of miR-34a or miR-145 significantly reduced expression of miR-34a target genes (*MDM4* and *SIRT1*) and miR-145 target genes (*ADAM17* and *NEDD9*), thus confirming that miR-34a and miR-145 mimics were functional in hESCs (Supplementary Fig. 7a, b). RT-qPCR and immunoblot analyses further showed that overexpression of miR-34a or miR-145 significantly reduced expression of the PATFs (*NANOG*, *OCT4*, and *SOX2*) at both mRNA and protein levels (Fig. 5h, i). The reduction in PATFs caused by ectopic expression of miR-34a or miR-145 was accompanied by upregulated expression of the LRTFs *Brachyury*, *GSC*, *MIXL1*, *SOX17*, *SOX1*, and *PAX6* (Fig. 6b, d), as well as PAX6, Brachyury, and SOX17 proteins (Fig. 6e). We then examined whether the expression of *circBIRC6* was capable of rescuing the loss of pluripotency and/or in vitro differentiation caused by miR-34a or miR-145 mimics in hESCs. RT-qPCR demonstrated that expression of *circBIRC6*-promoted expression of the PATFs, miR-34a, and miR-145-targeted genes (*MDM4*, *SIRT1*, *ADAM17*, and *NEDD9*) in hESCs ectopically expressing miR-34a and miR-145. Furthermore, expression of the LRTFs was suppressed (Fig. 6a–d; Supplementary Fig. 7a, b). Notably, these rescue effects were further enhanced by increasing the relative level of *circBIRC6* expression (circB (3 μg) in Fig. 6a–d. Furthermore, ICC staining showed that *circBIRC6* expression results in an increased number of NANOG^+^ and OCT4^+^ cells in hESCs ectopically expressing miR-34a or miR-145, and a decreased number of Brachyury^+^, SOX17^+^, and PAX6^+^ cells (Fig. 6e), suggesting that *circBIRC6* suppresses miR-34a-mediated and miR-145-mediated hESCs differentiation. Expression of *circBIRC6*, with mutations in miR-34a and miR-145 binding sites (circB4m), failed to rescue miR-34a-mediated and miR-145-mediated in vitro differentiation and the decrease of miR-34a-targeted and miR-145-targeted gene expression levels, further confirming that miRNA–*circBIRC6* interactions are necessary for *circBIRC6* function (Fig. 6a–d). Northern blotting using probes recognizing both *circBIRC6* and its linear precursors confirmed that *circBIRC6*, but not its linear precursors, was the major isoform expressed in hESC H9 transfected with circB or circB4m minigenes, supporting the idea that the rescue effects were related to *circBIRC6* expression (Supplementary Fig. 7c). In addition, RT-qPCR showed that expression levels of miR-34a-targeted and miR-145-targeted genes (*MDM4*, *SIRT1*, *ADAM17*, and *NEDD9*) were downregulated in H9-shcircB and rescued by re-expressing *circBIRC6* in H9-circB-res, supporting the notion that *circ* *BIRC6* acts as an miRNA sponge (Supplementary Fig. 7d). Collectively, our results support the conclusion that *circBIRC6* promotes pluripotency maintenance in hESCs by reducing the activity of miR-34a and miR-145.

### ESRP1 promotes the generation of circ*BIRC6* in hESCs

**Fig. 7:**
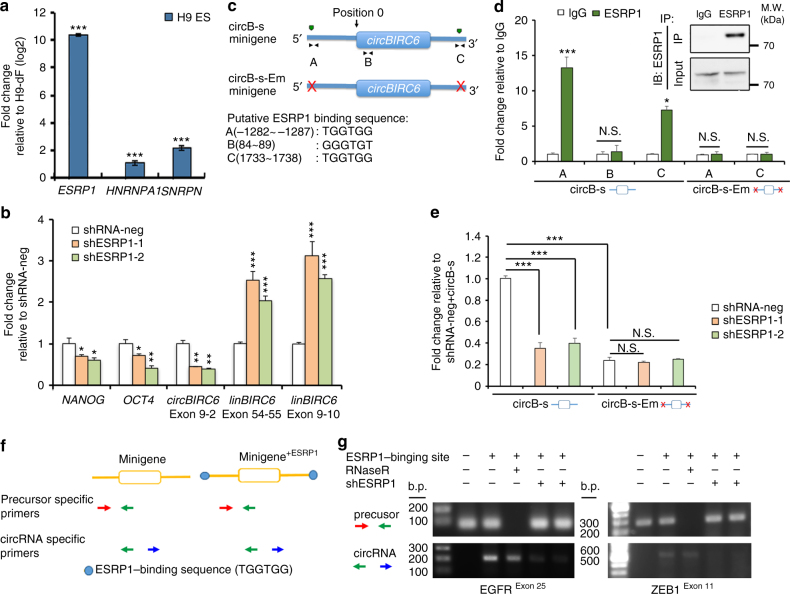
ESRP1 promotes the biogenesis of circ*BIRC6* in hESCs. **a** RT-qPCR analysis of the splicing factors (as indicated) in H9 hESCs. **b** RT-qPCR analysis of the expression of *circBIRC6* (exon 9–2), linear *BIRC6* (linBIRC6 exon 9–10 and exon 54–55), *NANOG*, and *OCT4* in ESRP1-knockdown hESCs (shESRP-1 and shESRP-2). **c** Schematic illustrating the putative ESRP1-binding sites on the flanking introns in the circB-s minigene. The 5′ terminus of the circular exons of *circBIRC6* was defined as position 0. Putative ESRP1-binding sites A, B, and C are located in the intron at the 5′ terminus of the *circBIRC6* exon (position: −1282 to −1287), within the *circBIRC6* exon (position: 84–89), and on the intron at the 3′ terminus of the *circBIRC6* exon (position: 1733–1738). **d** RIP analysis of ESRP1-binding to circB-s and circB-s-Em minigenes in hESCs. Bound complexes were pulled-down using an antibody against ESRP1. RT-qPCR was then used to measure circB-s binding to ESRP1. Values were normalized to the level of background RIP, as detected by an IgG isotype control. Inserted panel: Immunoblotting (IB) of immunoprecipitated (IP) ESRP1 protein. **e** RT-qPCR analysis of the expression of *circBIRC6* relative to *GAPDH* in hESCs. Cells were co-transfected with shRNA that target ESRP1 and a *circBIRC6* minigene (circB-s), or *circBIRC6* minigene containing deleted ESRP1-binding sites (circB-s-Em). **f** Schematic showing sites of insertion of ESRP1-binding sequence and the location of primers used for detecting the two minigenes (EGFR and ZEB1). **g** RT-PCR analysis using circRNA-specific or precursor specific primers on RNA from hESCs that were transfected with the indicated minigenes, with or without shRNA-mediated disruption of ESRP1. Quantitative data from three independent experiments is presented as mean ± SD (error bars). *P*-values were determined by two-tailed two-sample *t*-tests (*P < 0.05; **P < 0.01; ***P < 0.001)

As splicing factors (SFs) have been shown to regulate cell-type-specific circRNA expression^5, 10^, and we hypothesized that hESC-associated SF may be involved in generating *circBIRC6* in hESCs. Using microarray analyses, we identified three SFs, *ESRP1*, *SNRPN*, and *HNRNPA1*, that were enriched (fold change > 10) in hESC/iPSCs compared with human fibroblasts (Supplementary Data 3). RT-qPCR analyses further confirmed that these SFs were highly expressed in hESCs, with *ESRP1* being the highest expressed, when compared to the differentiated derivatives (H9-dF) (Fig. 7a), suggesting their expression may be associated with hESC pluripotency. To examine whether *ESRP1* is involved in *circBIRC6* generation in hESCs, we disrupted ESRP1 expression using two different shRNA variants (Supplementary Fig. 8) and investigated the effects of ESRP1-knockdown on *circBIRC6* expression. RT-qPCR analyses showed that ESRP1-knockdown resulted in a significant reduction of *circBIRC6* expression in hESCs, but increased the expression of linear *BIRC6* isoform (Fig. 7b). Notably, ESRP1 disruption also resulted in downregulation of the PATFs, *NANOG* and *OCT4*, in hESCs (Fig. 7b). It has been demonstrated that SFs promote circRNA generation by directly binding to recognition motifs in the introns flanking circRNA-forming exons^5, 10^. To examine whether ESRP1-binding sequences are critical for *circBIRC6* formation, we searched for ESRP1-binding sequences (GGT-rich)^28, 29^ in *circBIRC6* and its flanking introns, and identified three putative ESRP1-binding sites (Fig. 7c). Next, we generated two short *circBIRC6* minigenes, circB-s and circB-s-Em. The circB-s minigene contains putative ESRP1-binging sites on both flanking introns that are preserved, but in contrast to the *circBIRC6* minigene, the inversely inserted 5′ intron was removed to exclude the involvement of complementary sequences (Fig. 7c). The circB-s-Em minigene is similar to circB-s, but the putative ESRP1 sites have been deleted from the flanking introns. RIP analysis demonstrated the clear interaction between ESRP1 and circB-s, but not circB-s-Em (Fig. 7d), suggesting that ESRP1 indeed binds to the putative binding sites on the flanking introns. We then expressed circB-s in ESRP1 disrupted or control hESCs, and found that the circB-s minigene produced lower levels of *circBIRC6* transcript in the absence of ESRP1, confirming that ESRP1 is critical for *circBIRC6* formation in hESCs (Fig. 7e). Next, we expressed the circB-s-Em minigene in control hESCs, and found that circB-s-Em produced significantly less *circBIRC6* compared with circB-s. This result suggested that the putative ESRP1-binding motifs on flanking introns are critical for *circBIRC6* production (Fig. 7e). Notably, disruption of ESRP1 expression in circB-s-Em-transduced hESCs did not further decrease *circBIRC6* transcript levels. To extend these observations, we investigated whether exons that do not normally form circRNAs (based on the circRNA collection in circBase^30^) could be made competent to generate circRNAs in hESCs by inserting ESRP1-binding sites into the flanking introns. We selected one exon with a length that was compatible to *circBIRC6* (exon 11 of ZEB1) and another exon with shorter length (exon 25 of EGFR) to generate the minigene constructs. The minigenes were constructed to express regions containing the exons and the endogenous flanking introns (Fig. 7f) and were subsequently expressed in hESCs. Our RT-PCR results revealed that the expression of the aforementioned minigenes, harboring introduced ESRP1-binding motifs, produced circRNAs, whereas the minigenes without the inserted ESRP1-binding motif did not yield circRNAs (Fig. 7g). Then, we disrupted ESRP1 expression in hESCs that also expressed the ESRP1-binding motif inserted minigenes containing exon 11 of ZEB1 or exon 25 of EGFR and analyzed circRNA expression by RT-PCR. Our analysis showed that the circRNA formation was severely compromised by disrupting the expression of ESRP1 in hESCs (Fig. 7g), suggesting that the flanking intronic sequences, which contain ESRP1-binding motifs, can induce ESRP1-dependent circularization from transcripts that are normally linearly spliced. Taken together, these experiments demonstrate that ESRP1 plays an important role in regulating the formation of *circBIRC6* in hESCs through binding to recognition sites in the introns flanking the circBIRC6-forming exons.

### NANOG and OCT4 regulate ESRP1 expression in hESCs

**Fig. 8:**
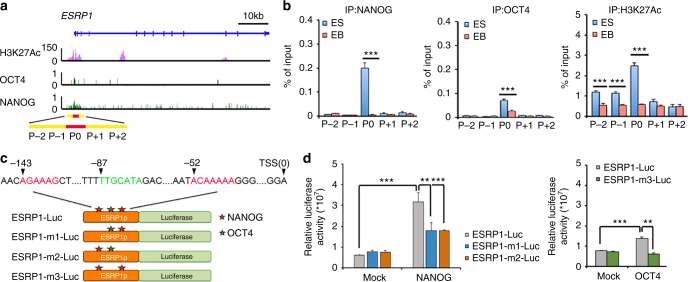
NANOG and OCT4 regulate ESRP1 expression in hESCs. **a** ENCODE ChIP-Seq data for NANOG and OCT4 occupancy, and H3K27Ac modifications were aligned to the *ESRP1* promoter, defined as −1 kb to +1 kb relative to the transcription start site. The *y* axis represents the intensity of ChIP-Seq reads. The highest NANOG and OCT4 binding peaks on the *ESRP1* promoter are labeled as P0 (red bars; Chr8: 95653024–95653560), and four flanking regions within −1 kb to +1 kb (yellow bars) of P0 are labeled P−1, P−2, P+1, and P+2. **b** ChIP fragments containing the NANOG and OCT4 binding peak, or its four flanking regions, were quantified in hESCs and embryoid bodies (EBs) by qPCR using primers specific for P0, P+1, P+2, P−1, and P−2, respectively (Supplementary Table 1), and normalized to the input genome used in ChIP. **c** Schematic illustrating luciferase reporters containing an *ESRP1* promoter (ESRP1-luc), ESRP1-luc with deleted NANOG binding sites (ESRP1-m1-luc and ESRP1-m2-luc), and ESRP1-luc with a deleted OCT4 binding site (ESRP1-m3-luc). **d** Luciferase reporter analysis showing the luciferase activity of ESRP1-luc, ESRP1-m1-luc, ESRP1-m2-luc, and ESRP1-m3-luc in 293 T cells ectopically expressing NANOG (left panel) or OCT4 (right panel). Quantitative data from three independent experiments is presented as mean ± SD (error bars). *P*-values were determined by two-tailed two-sample *t*-tests (*P < 0.05; **P < 0.01; ***P < 0.001)

As ESRP1 was highly expressed in human pluripotent stem cells (Fig. 7a; Supplementary Data 3), we hypothesized that the expression of ESRP1 may be directly regulated by PATFs. To explore this possibility, we first searched the ENCODE ChIP-seq database^31^ for PATFs that may occupy the *ESRP1* promoter. Our analysis showed that NANOG and OCT4 occupancy, and H3K27Ac histone modifications were enriched on the *ESRP1* promoter (Fig. 8a). ChIP-qPCR analyses further revealed that NANOG and OCT4 occupancy and H3K27Ac histone modifications were enriched in hESCs, but not in differentiated derivatives (Fig. 8b). Furthermore, a binding-motif analysis showed two putative binding sites for NANOG and one for OCT4 on the *ESRP1* promoter (Fig. 8c). Finally, to determine whether NANOG and OCT4 directly regulate ESRP1 expression, we created an *ESRP1* promoter-luciferase reporter (ESRP1-luc) construct and showed that its activity was increased by NANOG and OCT4 (Fig. 8d). This increase was attenuated by deletion of *ESRP1* promoter NANOG binding sites (ESRP1-m1-Luc and ESRP1-m2-Luc) or the OCT4 binding site (ESRP1-m3-Luc) (Fig. 8d). Taken together, these results demonstrate that NANOG and OCT4 bind to the *ESRP1* promoter to promote *ESRP1* expression in hESCs.

## Discussion

It is evident that large numbers of circRNAs exist in the human transcriptome, and accumulating evidence suggests that they may have important roles in regulating cellular function^6, 13, 14^. However, the role of circRNA in hESCs and the mechanism by which circRNAs regulate pluripotency has not been previously described. In this study, we identified a subset of circRNAs that are enriched in hESCs and demonstrated that two circRNAs, *circBIRC6* and *circCORO1C*, are functionally associated with the pluripotent state. Loss-of-function experiments demonstrated that compromised *circBIRC6* or *circCORO1C* expression impeded pluripotency and induced differentiation of hESCs. In addition, ectopic expression of *circBIRC6* or *circCORO1C* in hESCs-promoted pluripotency and enhanced the efficiency of iPSCs generation. Mechanistically, we found that *circBIRC6* suppressed miR-34a-mediated and miR-145-mediated differentiation by directly interacting with these miRNAs. Our data thus suggest that *circBIRC6* functions as a miRNA sponge to attenuate miRNA-mediated activity. Our study also demonstrates that ESRP1, a hESC-enriched SF, promotes *circBIRC6* synthesis through interactions with its flanking introns. Furthermore, by using ChIP-qPCR and reporter analyses, we found that the *ESRP1* expression is regulated by NANOG and OCT4 in hESCs. Our study suggests the existence of a regulatory network in hESCs, wherein coordinated interactions between PATFs and SFs modulate the expression of a distinct group of circRNAs, which can directly interact with miRNAs and influence the pluripotency state.

It was shown that circRNAs play roles in various biological processes, including neural development (*ciRS-7/CDR1as*), spermatogenesis (*SRY*), and cancer cell proliferation (*circFOXO3*, *circHIPK3*, and *circITCH*)^6, 12–14^. Here, we demonstrate that both *circBIRC6* and *circCORO1C* are functionally associated with the human pluripotency. Our miRNA-targeting analysis showed that *circBIRC6* harbors 431 targeting sites for 306 different miRNAs, suggesting that *circBIRC6* may act as a miRNA sponge. Among these 306 miRNAs, there are 70 with two binding sites on *circBIRC6*, 21 with three binding sites, and four with four binding sites (Supplementary Data 2). These results are in agreement with a previous report, which showed that no particular miRNA exhibited highly enriched binding to circRNAs, with the exceptions of *CDR1as*, *SRY*, and *ZNF91* ^32^. Furthermore, our results confirmed that *circBIRC6* can interact with and sequester at least two miRNAs, miR-34a and miR-145, to prevent in vitro differentiation. In addition, *circBIRC6* has binding sites for several other miRNAs, including let-7, miR-92, and miR-103 (Supplementary Data 2), which have previously been reported to have functional roles in early-lineage differentiation. Therefore, in line with recent studies^12–14^, our results support the idea that *circBIRC6* can serve as a miRNA sponge for multiple miRNAs to regulate gene expression.

Our results suggest that, unlike *circBIRC6*, *circCORO1C* likely contributes to controlling hESC pluripotency through a mechanism other than sequestration of miRNA(s). C*ircCORO1C* does not associate with AGO2, which is a critical component for the miRNA-sponge role of circRNAs. Instead, we considered that *circCORO1C* may potentially function by regulating its linear isoform through competition with pre-mRNA synthesis, as recently demonstrated for *circMbl* ^10^. However, disrupting *circCORO1C* expression did not affect the expression of its linear counterpart (Fig. 2b), thus precluding this possibility. Alternatively, *circCORO1C* may regulate hESC pluripotency through interactions with proteins, for example, *circFoxo3* can inhibit cell-cycle progression by trapping p21 and CDK2 in the cytoplasm^33^. Further experiments will be necessary to clarify whether this mechanism, or another, governs *circCORO1C* activity.

Although our data indicate that *circBIRC6* and *circCORO1C* are functionally associated with pluripotency, we found that expression of either *circBIRC6* or *circCORO1C* in human fibroblasts was not sufficient to reactivate the pluripotency program and effect iPSC formation. On the other hand, co-expression of either *circBIRC6* or *circCORO1C* with OSKM in human fibroblasts potentiated iPSC formation. One likely explanation for these findings is that *circBIRC6* and *circCORO1C* alone may not be sufficient to reactivate all the major pathways of early stage pluripotency reprogramming^34^, and thus cannot initiate the reprogramming process in somatic cells. Nevertheless, we found that these circRNAs enhance OSKM-initiated reprogramming. It is possible that *circBIRC6* may contribute to improving reprogramming efficiency by blocking P53, a major roadblock of pluripotency reprogramming^35^, through upregulation of the P53 suppresser MDM2, which is a common target of miR-145^36^. Similar to miR-145, miR-34a has also been described as a suppressive factor for reprogramming, and inhibition of miR-34a expression has been shown to enhance reprogramming efficiency^18^. Although we did not find that pluripotency reprograming is possible using *circBIRC6* or *circCORO1C* alone, it remains conceivable that in the future, iPSCs may be created using combinations of circRNAs that target and regulate miRNAs to control the major reprogramming pathways, thus allowing circRNAs to replace transcription factors for iPSC generation^37^.

The differential expression of *circBIRC6* between human fibroblasts and hESCs suggests that biogenesis of *circBIRC6* is regulated in a cell-type-specific manner, and that hESCs possess specific elements involved in circRNA biogenesis. Indeed, we found that ectopic expression of a *circBIRC6* minigene in hESCs, or their differentiated derivatives, promoted the generation of *circBIRC6* and its linear precursor (Figs. 3b and 4b). However, the linear precursor of *circBIRC6* was less efficiently converted into *circBIRC6* in hESC-derived fibroblasts than in hESCs, suggesting that *circBIRC6* biogenesis may be regulated in a cell-type-dependent manner. Furthermore, we identified several splicing factors that are highly enriched in hESCs, and demonstrated that ESRP1 is involved in *circBIRC6* biogenesis. These observations support the idea that splicing factors can contribute to cell-type-specific circRNA biogenesis^5, 10^. We also showed that the pluripotency-associated factors OCT4 and NANOG could directly regulate the expression of ESRP1, providing evidence to further support the interpretation that the ESRP1–*circBIRC6* axis is a pluripotency-associated regulatory pathway. Although we showed that ESRP1 promoted *circBIRC6* formation, ESRP1-knockdown only reduced *circBIRC6* expression by ~50%. This incomplete suppression may be attributable to the redundant effects of ESRP2, a close family member of ESRP1 that recognizes a similar binding motif. We cannot rule out the possibility that *circBIRC6* biogenesis is regulated by multiple hESC-enriched SFs, as was shown in other systems^38^. Future studies will be required to elucidate how other hESC-enriched SFs function to control human pluripotency.

We have shown that altered ESRP1 expression may have profound impact on *circBIRC6* biogenesis. Moreover, our mechanistic studies revealed that deletion of the ESRP1-binding motifs in the introns flanking circBIRC6 resulted in a dramatic reduction of circBIRC6 generation. However, insertion of ESRP1-binding motifs into introns, which flank exons that normally do not form circRNA, induced circRNA formation from these exons. Altogether, these results suggest that ESRP1 may directly regulate back-splicing to promote *circBIRC6* generation in hESCs through interaction with the putative binding motifs on both flanking introns of *circBIRC6*.

SFs are emerging regulators of pluripotency. For instance, MBNL promotes in vitro differentiation of hESCs through regulation of FOXP1 isoform-switching^39^. In contrast, the SR-related SFs SON and SFRS2 contribute to the maintenance of pluripotency by regulating the splicing of OCT4^40^ and methyl-CpG binding domain protein (MBD2)^41^, respectively. In this study, we showed that disruption of ESRP1 reduced the expression of NANOG and OCT4, suggesting that ESRP1 indirectly regulates pluripotency by controlling *circBIRC6* biogenesis. However, we cannot exclude that ESRP1, like SFRS2, directly regulates OCT4 and NANOG to promote pluripotency^41^. If this is the case, ESRP1 and OCT4 or NANOG could form a positive-feedback loop to reciprocally control the level and function of themselves and circRNAs, and thereby prevent loss of pluripotency in hESCs (see model in Supplementary Fig. 9). Nevertheless, these results suggest that, in addition to regulating the core transcription factor network in pluripotency, SFs could generate another layer of regulation by promoting circRNA synthesis or inducing pluripotency-associated genes.

## Methods

### Cell culture

Mouse (strain ICR) embryonic fibroblasts (MEFs) and human fibroblasts (HFs) were expanded in Dulbecco’s Modified Eagle Medium (DMEM) supplemented with 10% fetal bovine serum (FBS; Hyclone), 1% non-essential amino acids (Invitrogen), and 2 mM l-glutamine (Invitrogen). Mitomycin C (Sigma) was used to inactivate MEFs at P4 for generation of feeder cells. Human ESCs H9 and iPSCs were grown on MEF feeder layers (2 × 10^4^ per cm^2^) in DMEM/F12 medium (Invitrogen) plus 20% Knockout Serum Replacement (Invitrogen), 1 mM l-glutamine, and 4 ng ml^−1^ basic fibroblast growth factor (bFGF; BD Biosciences). For in vitro differentiation, hESC colonies were detached by treating cells with 1 mg ml^−1^ dispase (Invitrogen) for 20 min at 37 °C and then transferring detached cells to ultra-low-attachment plates (Corning) to allow formation of embryoid bodies (EBs). The medium for EB culture was changed daily for 4 d using the same medium for culturing hESCs, and then replaced with MEF medium for further differentiation. The hESC H9 are obtained from WiCell Research institute and HFs are obtained from ATCC. Mycoplasma contamination was monthly examined by EZ-PCR mycoplasma test kit (Biological Industries). All animal experiments were performed under protocols approved by the Academia Sinica Institutional Animal Care and Utilization Committee.

### RNA extraction and qRT-PCR

Total RNA was isolated from cells using the TRIzol reagent (Invitrogen) according to the manufacturer’s instructions. Purified RNA was treated with DNase I (Qiagen) to remove genome contamination and then was reverse transcribed using a Superscript II reverse transcriptase kit (Invitrogen) to generate a cDNA library. PCR products were amplified for 35 cycles using GoTag Mastermix (Promega), and RT-qPCR analyses were performed in triplicate using a KAPA SYBR Fast Kit (KAPA). All primers used are listed in Supplementary Table 1.

### RNaseR treatment

Total RNA (10 μg) from hESC H9 cells was incubated with 40 U RNaseR at 37 °C for 1 h in 1× buffer provided with the enzyme (Epicentre). RNA transcripts resistant to RNaseR treatment were precipitated with ethanol and resuspended in RNase-free H_2_O for the indicated analyses.

### Identification and validation of circRNAs

Step 1: A total of 61 circRNAs with greater than 40 reads supporting a circular junction were selected from a database previously described by Memczak et al.^6^ Step 2: Circular junctions of the 61 circRNAs selected in step 1 were validated by sequential RNaseR treatment, RT-PCR, and sequencing assays; 20 circRNAs were validated. Step 3: hESC-enriched circRNAs were selected from the 20 circRNAs validated in step 2 using RT-qPCR assays (>0.1% GAPDH); 11 circRNAs were selected. Step 4: circRNAs highly associated with the pluripotent state were selected using RT-qPCR assays (fold change > 4 in reprogramming and differentiation); three circRNAs were selected. Step 5: Three circRNAs whose expression was highly associated with the pluripotent state (fold change > 4 in reprogramming and differentiation) were subjected to further functional tests (gain-of-function and loss-of-function assays). Full length of DNA electrophoresis of validated circRNAs and their linear counterpart shown in Fig. 1 are provided in Supplementary Fig. 10.

### Expression vectors and miRNA mimics

For circRNA-expressing FUW vectors, full-length circRNAs without intervening introns were cloned from a cDNA library of H9 hESCs. The endogenous 5′-flanking and 3′-flanking 1 kb introns were cloned from the genome and fused to the corresponding ends of the circRNA. Notably, the exon–intron boundaries and canonical splicing sites (GT-AG) at the 5′ and 3′ termini of circRNAs were preserved for correct splicing. The most upstream 1 kb sequence of the 5′-flanking intron was copied and inversely inserted downstream of the 3′-flanking intron. To generate the circB-s minigene vector, the inversely inserted 5′-flanking intron at the 3′ terminus of the circBIRC6 minigene was removed by restriction enzyme digestion. For the circB-s-Em minigene vector (circB-s minigene with deleted ESRP1-binding sites), the region spanning between two ESRP1-binding sites at the 5′ and 3′ termini of the circB-s minigene vector was cloned by PCR and fused into the FUW vector. For minigenes expressing EGFR Exon 25 and ZEB1 Exon 11, the targeted exons with endogenous 5′-flanking and 3′-flanking 0.5 kb introns were cloned from the genome and fused into the FUW vector. The ESRP1-binding sequences were added to the 5′ and 3′ termini of EGFR Exon 25 and ZEB1 Exon 11 minigenes by PCR using primers that included ESRP1-binding sequences at the 5′ termini. For shRNA-expressing vectors, shRNA oligonucleotides specifically targeting the circular junction were synthesized (Genedragon), cloned into the pLKO vector according to the standard annealing protocol provided by the RNAi core (Academia Sinica, Taiwan), and verified by sequencing. Targeting sequences are listed in Supplementary Table 1. Lentiviral particles carrying circRNAs or shRNA or minigenes were generated in HEK293T cells using standard procedures. hESCs were infected with lentivirus and then cultured in standard hESC medium. The hESCs were then harvested for indicated analyses 3 days after infection. The miR-34a and miR-145 mimics or scrambled oligonucleotides (MDBio) were transfected into hESCs using Lipofetamine RNAiMAX (Thermo) at a final concentration of 20 nM. Transfected cells were harvested for indicated analyses 3 days after transfection.

### Immunofluorescence staining and cell counting

Human ESCs and iPSCs were cultured on cover glass and fixed by incubating with 4% paraformaldehyde for 20 min at room temperature (RT). Fixed cells were then washed with PBS and permeabilized using a non-ionic detergent (0.1% Triton X-100 and 0.2% Tween-20) in PBS for 40 min at RT. Permeabilized cells were blocked by incubating with 2% goat serum (Invitrogen) for 1 h, washed with PBS containing 0.01% Tween-20 (PBST), and incubated with primary antibody. Primary antibodies used included anti-SOX17 (1:200; R&D Systems; AF1924), anti-PAX6 (1:500; Abcam; Ab5790), anti-NANOG (1:50; Abcam; Ab21624), anti-OCT4 (1:1000; Millipore; MAB4401), and anti-Brachyury (1:50; R&D Systems; AF2085). Cells were then washed with PBST and incubated with the appropriate fluorescein-conjugated secondary antibody. Stained samples were mounted using Vectashield H-1200 mounting media (Vector Laboratories), and images were captured using a fluorescence microscope (Leica). Positive signals in IF images were processed and counted using Image J with a consistent intensity threshold.

### AP staining

Cells on regular Petri dishes were fixed by incubating with 4% paraformaldehyde for 20 min at RT and then washed with PBS. Fixed cells were then incubated at RT with an AP substrate working solution, according to the instructions of the Blue Alkaline Phosphatase Substrate Kit (Vector Laboratories), to allow color development. The number of AP^+^ colonies was counted using Image J with a consistent intensity threshold.

### FACS analysis

Cells were detached with trypsin, washed with PBS, fixed with 1% paraformaldehyde and then permeabilized with 0.1% Triton X-100 in PBS. Fixed/permeabilized cells (1 × 10^6^) were suspended in 100 μl PBS containing 1% bovine serum albumen (BSA) FACSCalibur (BD Biosciences) was used to count GFP^+^ cells, and collected data were analyzed with Cell Quest software (BD Biosciences).

### Western blotting

Protein was extracted with RIPA buffer and quantified using a BCA protein assay kit (Pierce). For sodium dodecyl sulfate-polyacrylamide gel electrophoresis (SDS-PAGE), 20 μg of protein from each sample was resolved on 10% gels and electro-transferred to a nitrocellulose membrane. After blocking non-specific binding by incubating in 3% non-fat milk in PBST for 1 h, at RT, membranes were incubated at 4 °C overnight with diluted primary antibody. The primary antibodies used were anti-OCT4 (1:1000; Millipore; MAB4401), anti-NANOG (1:1000; Abcam; Ab21624), anti-SOX2(1:2000; Millipore; AB5603), anti-AGO2 (1:1000; Abcam; ab57113), anti-ESRP1 (Novus; NBP1-82201), and mouse anti-β-actin (1:5000; Sigma; A5441). After washing with PBST, membranes were incubated with the appropriate horseradish peroxidase (HRP)-conjugated secondary antibody for 1 h, at RT. Signals were developed using Supersignal West Femto Substrate (Pierce) and recorded with an LAS-4000 luminescence image analyzer (Fujifilm). Full scans of the western blots shown in Fig. 5 are provided in Supplementary Fig. 11.

### CRISPR-Cas9n-mediated genome editing

Homologous recombination between the first exon of OCT4 and donor vector through the homologous left arm (HL) and right arm (HR) was promoted by co-transfecting hESCs with donor vectors containing GFP, EF1α-RFP (RFP driven by an EF1α promoter), and puromycin (PURO) together with two guide RNAs (sequences in Supplementary Table 1). EF1α-RFP and PURO were subsequently removed using Cre protein after selection of transgenic hESC clones for expansion.

### Biotinylated miRNA mimic capture

The biotinylated miRNA mimic pull-down assay was performed as described by Lal et al.^42^ In brief, 3′ end-biotinylated miR-34a and miR-145 mimics or scrambled oligonucleotides (MDBio) were transfected into hESCs using Lipofetamine RNAiMAX (Thermo) at a final concentration of 20 nM. After 1 day, the molecules interacting with biotinylated miRNA mimics were pulled-down by incubating the cell lysates with streptavidin-coated magnetic beads (Life Technologies). The abundance of *circBIRC6* or *circCORO1C* in bound fractions was evaluated by RT-qPCR analysis.

### RNA immunoprecipitation

RNA immunoprecipitation (RIP) assays were performed with using a Magna RIP Kit (Millipore) following the manufacturer’s instructions. In brief, 2 × 10^7^ H9 hESCs were UV-crosslinked at 600 mj cm^−2^ and lysed in 100 μl RIP lysis buffer containing a proteinase inhibitor cocktail (Roche) and RNase inhibitor (Promega). Lysate was treated with DNase I (Roche) at 37 °C for 10 min and centrifuged at 12,000×*g* for 30 m. The lysate was then diluted with 900 μl RIP immunoprecipitation buffer and incubated for 3 h with 5 μg anti-ESRP1 (Novus, NBP1-82201) or 5 μg anti-AGO2 (Abcam, ab57113) antibodies that were pre-bound on magnetic beads. A 10-μl aliquot of the RIP mixture was saved as input. Beads were then washed six times with RIP wash buffer. A portion (20%) of the immunoprecipitate was saved for western blot analysis and the remainder (80%) was treated with proteinase K at 37 °C for 30 min. RNA was extracted using TRIzol regent (Invitrogen) according to the manufacturer’s instructions.

### Chromatin immunoprecipitation assay

Chromatin immunoprecipitation (ChIP) assays were performed as follows^22^: briefly, hESCs H9 and their differentiated derivate (H9-EB) were treated with 1% (v/v) formaldehyde for 8 min at room temperature, and quenched with 125 mM glycine. The crosslinked chromatin was sonicated to an average size of 100–150 bp. Each ChIP reaction contained sonicated chromatin from 5 × 10^5^ cells and 2 μg antibody (anti-OCT4 antibody, Millipore, MAB4401; anti-NANOG antibody, Abcam, Ab21624; anti-H3K27Ac, Abcam, Ab4729). The isolated DNA was purified using a QIAquick PCR purification kit (Qiagen). NANOG and OCT4 binding to the *ESRP1* promoter was quantified by qPCR. Primers are listed in Supplementary Table 1. Enrichment (% of Input) was determined as (ChIP DNA/input DNA) × 100%. Each experiment was repeated at least three times, with each replicate producing a similar pattern. The results are expressed as mean ± SD of three qPCR replicates.

### Statistical analyses

All quantitative data were obtained from three independent biologically replicated experiments and are presented as means ± SDs (error bars). *P*-values < 0.05, determined by two-tailed two-sample *t*-tests (unless otherwise indicated), were considered statistically significant.

### Data availability

The microarray data for the splicing factors expression reported in Supplementary Data 3 are available in Gene Expression Omnibus with accession numbers GSE28406 (HF and iCFB50) and GSE19964 (H9). All relevant data are available from the authors.

## Electronic supplementary material

Supplementary Information

Description of Additional Supplementary Files

Supplementary Data 1

Supplementary Data 2

Supplementary Data 3
