# Supplementary material for: The circular RNA circBIRC6 participates in the molecular circuitry controlling human pluripotency
Source: Nat Commun. 2017 Oct 27;8:1149. doi: 10.1038/s41467-017-01216-w (PMC5658440; doi:10.1038/s41467-017-01216-w)
Supplement: Supplementary file 1 — Supplementary Information [file 41467_2017_1216_MOESM1_ESM.pdf]

Supplementary Figures:

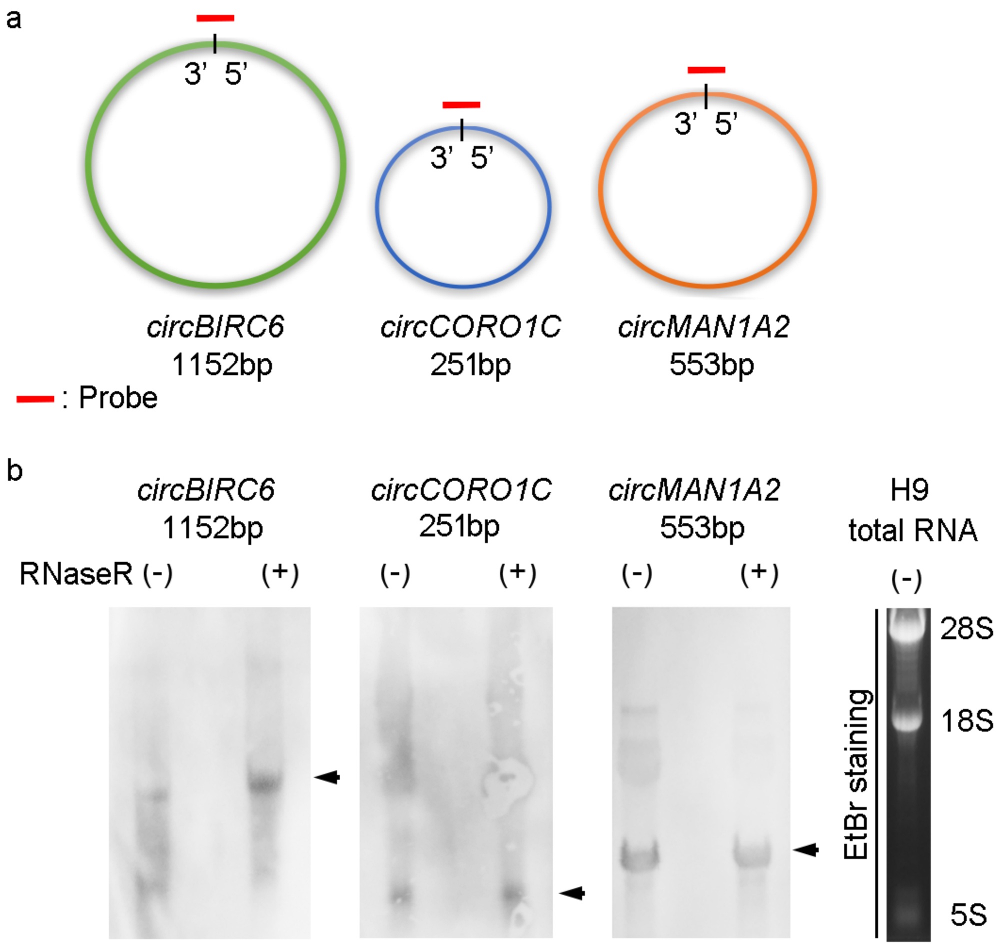

Supplementary Figure 1: Northern blotting analysis of circRNA expression in RNaseR-treated total RNA from hESC H9. (a) Schematic illustrates the size of *circBIRC6, circCORO1C*, and *circMAN1A2* and the circular junction region targeted by RNA probes. Divergent primers used for probe generation are listed in Supplementary table 1. (b) Northern blotting analysis shows the hybridization of probes to the circular junction of the indicated circRNAs in RNaseR-treated hESC total RNA. Arrow indicates the hybridization signal of the RNA probes. EtBr staining of rRNA 28S (5070 nt), 18S(1869nt), and 5S (121nt) are used as size standards.

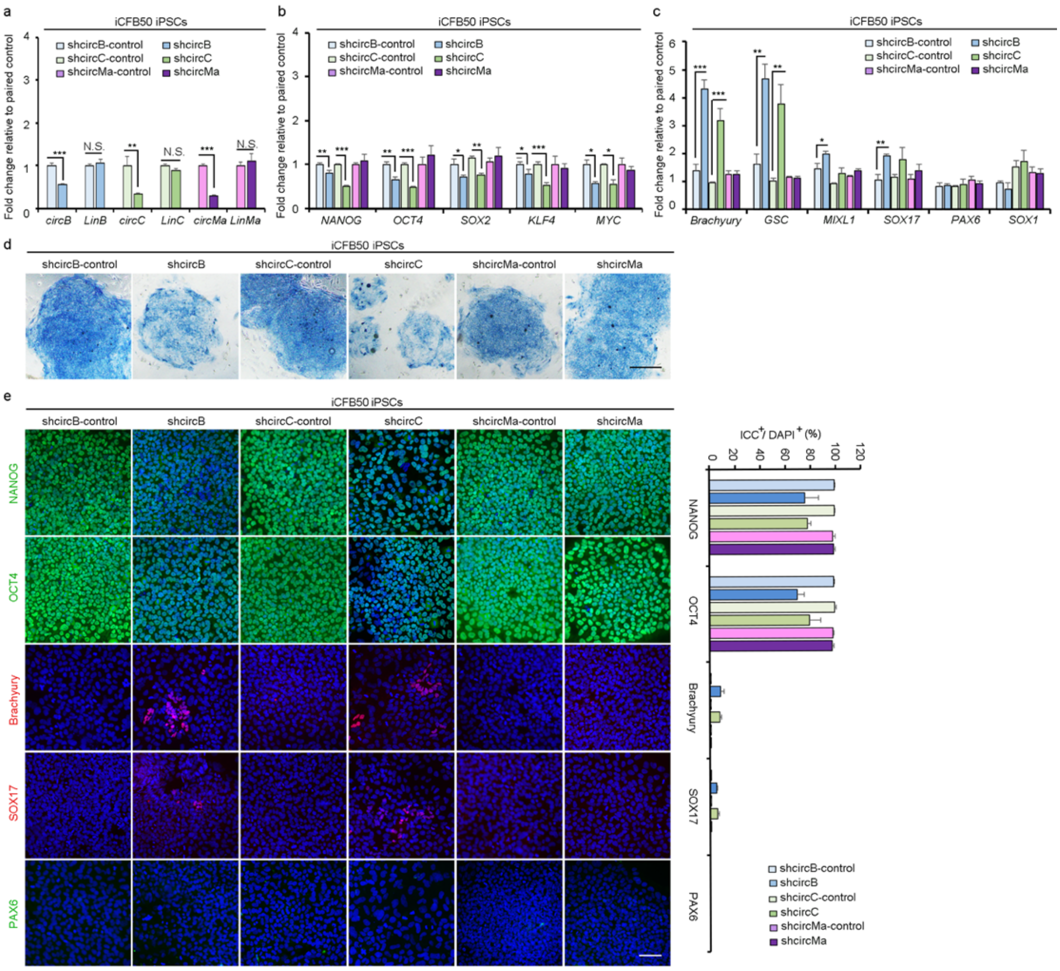

Supplementary Figure 2: Disruption of circ*BIRC6* or circ*CORO1C* in iPSCs impairs pluripotency maintenance. (a to c) iPSCs were transfected with shcircB, shcircC, shcircMa or paired control virus. Total RNA was isolated 3 days post transduction and analyzed by RT-qPCR to determine the expression levels of (a) *circBIRC6* (circB), *circCORO1C* (circC), *circMAN1A2* (circMA), their linear counterparts (LinB, LinC and LinMa), as well as the indicated (b) pluripotency- and (c) lineage-associated genes. (d) AP staining of shcircB, shcircC and shcircMa-transduced iCFB50 iPSCs. Scale bar: 100 μm. (e) ICC analysis of shcircB, shcircC or shcircMa-transduced iCFB50 iPSCs 3 days post virus infection using antibodies against the indicated pluripotency and lineage markers as indicated. Corresponding bar graphs show quantitative analysis of the percentage of cells expressing pluripotency- or lineage-associated markers. Mean ± SD were determined from quantitative data obtained from three independent experiments (*$P < 0.05$, **$P < 0.01$, and ***$P < 0.001$; N.S.: non-significant; two-tailed two-sample *t*-test).

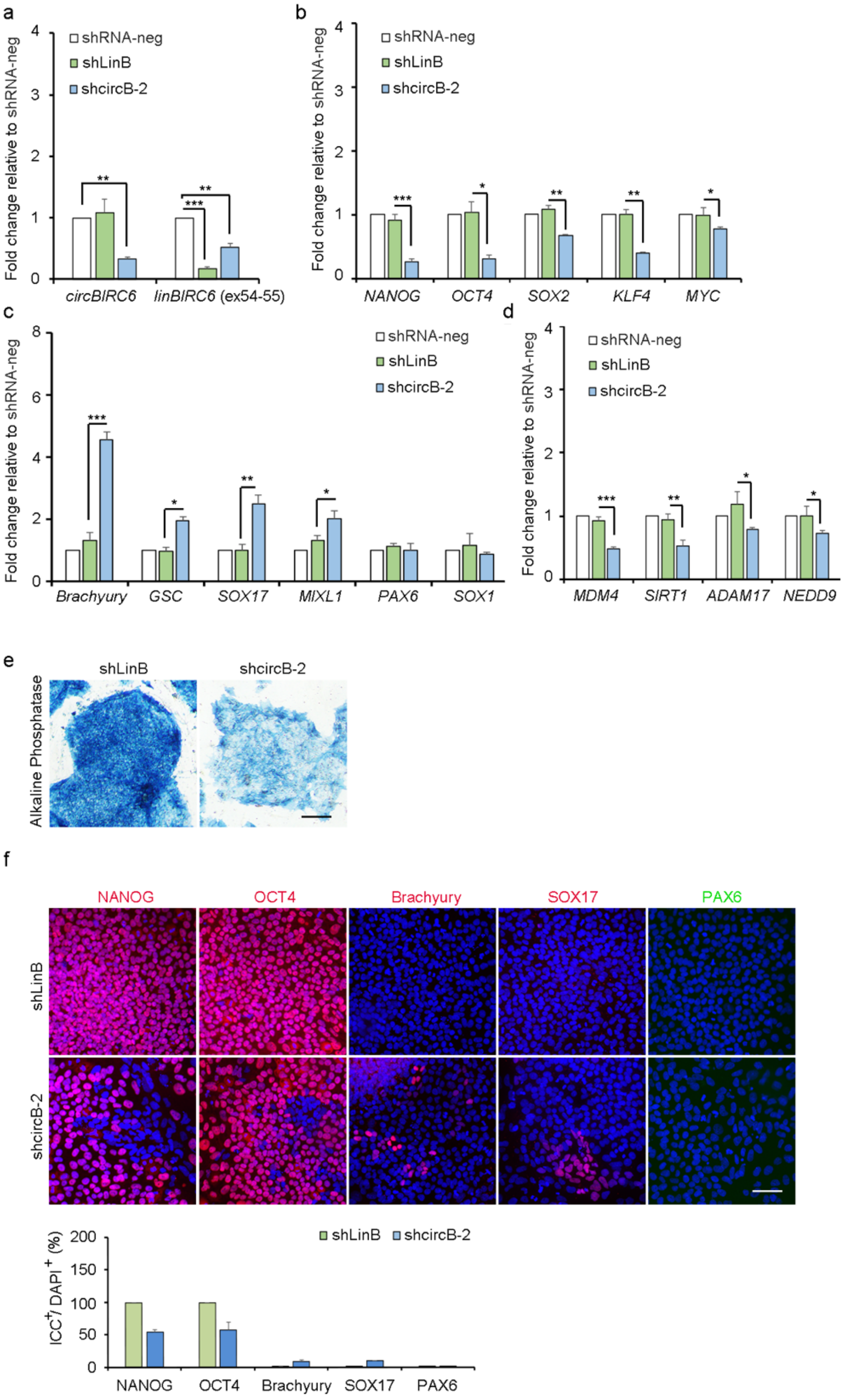

Supplementary Figure 3: Knockdown of circ*BIRC6*, but not linear *BIRC6*, impairs pluripotency maintenance in hESCs. (a to c) iPSCs were transfected with shcircB-2 or shLinB virus. Total RNA was isolated 3 days post transduction and analyzed by RT-qPCR to determine the expression levels of (a) *circBIRC6*, linear *BIRC6* (ex54-55), the indicated (b) pluripotency- and (c) lineage-associated genes, and (d) miR-34a and miR-145 targeting

genes. (e) AP staining of shcircB-2 and shLinB transduced H9 hESCs. Scale bar: 100 μm. (f) ICC analysis of shcircB-2 and shLinB transduced H9 hESCs 3 days post virus infection using antibodies against the indicated pluripotency and lineage markers as indicated. Bar graphs show quantitative analysis of the percentage of cells expressing the indicated pluripotency- or lineage-associated markers. Mean ± SD were determined from quantitative data obtained from three independent experiments (*$P < 0.05$, **$P < 0.01$, and ***$P < 0.001$; two-tailed two-sample *t*-test).

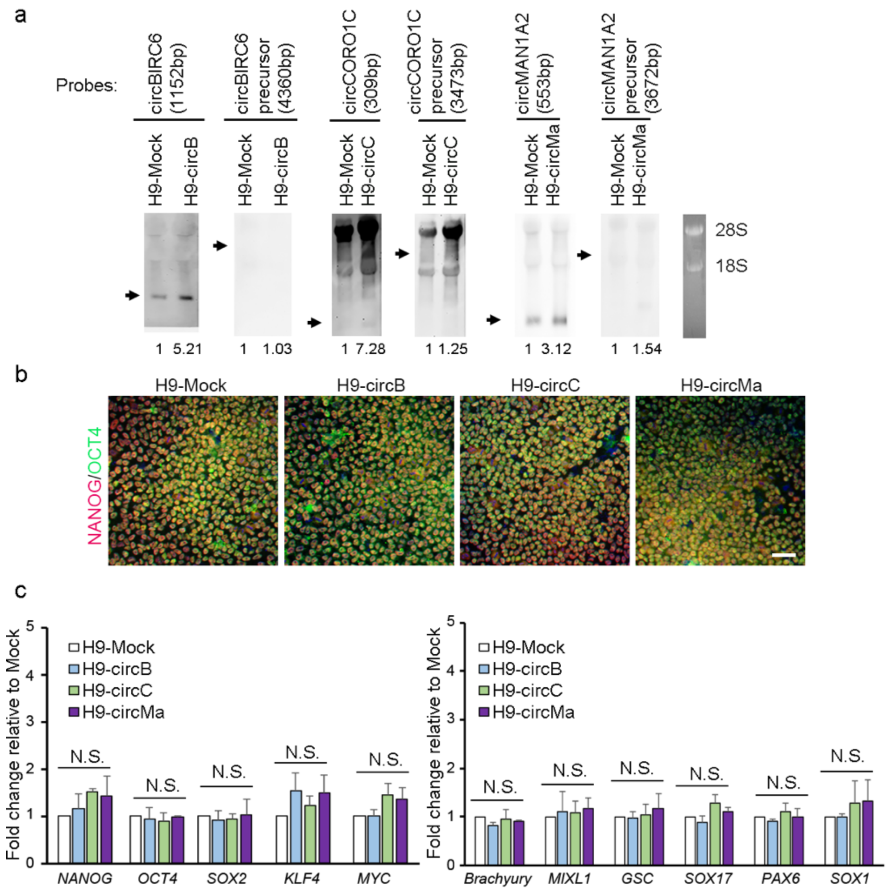

Supplementary Figure 4: Ectopic expression of *circBIRC6, circCORO1C* or *circMAN1A2* in hESCs does not cause a loss of pluripotency. (a) Northern blotting analysis shows the hybridization of probes to the circular junction of circRNA (circB, circC and circMa) or to the specific exon-intron junction of circRNA precursors (pre-circB, pre-circC and pre-circMa). Total RNA was probed from hESCs ectopically expressing *circBIRC6* (H9-circB), *circCORO1C* (H9-circC) or *circMAN1A2* (H9-circMa) expression. Arrows indicate the hybridization signal for each RNA probe. EtBr staining of 28S (5070 nt) and 18S(1869nt) rRNA are used as size standards. Quantification of RNA level is shown below each blot as the ratio of signal intensities compared to H9-Mock (b) ICC staining of NANOG and OCT4 expression in hESC expressing *circBIRC6* (H9-circB), *circCORO1C* (H9-circC) and *circMAN1A2* (H9-circMa). (Scale bar: 20 μm). (c) RT-qPCR analysis of expression of pluripotency- and lineage-associated genes (as indicated) in H9-circB, H9-circC and H9-circMa expressing cells. Mean ± SD were determined from quantitative data obtained from three independent experiments (N.S.: non-significant; two-tailed two-sample *t*-test).

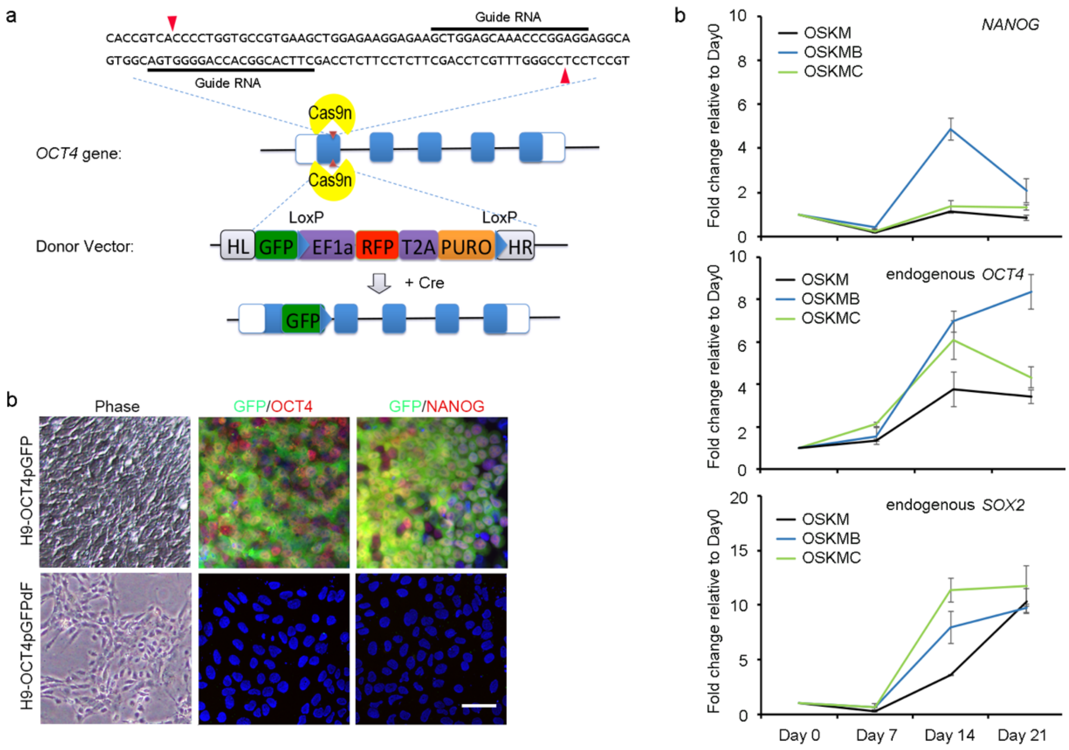

Supplementary Figure 5: The generation and characterization of OCT4-GFP reporter hESCs (H9-OCT4pGFP). (a) Schematic illustrating the CRISPR-mediated knock-in strategy used to generate H9-OCT4pGFP cells. Donor vectors contained GFP, EF1α-RFP (RFP driven by the EF1α promoter) and puromycin (PURO) were co-transfected with Cas9 nickase (Cas9n) and two guide RNAs (For sequences, see table S4) to promote homologous recombination between the first exon of OCT4 (red triangle) and donor vector through homologous left arm (HL) and right arm (HR). The EF1α-RFP and PURO were subsequently removed by Cre protein in transgenic hESC clones that were selected for expansion. (b) Immunofluorescence staining analysis of the pluripotency-associated markers NANOG and OCT4 in H9-OCT4pGFP cells and H9-OCTpGFP-derived fibroblasts (H9-OCTpGFPdF). Nuclei were stained with DAPI (blue). Scale bar: 20 μm. Note that OCT4-GFP is co-localized with endogenous OCT4 or NANOG (red) in hESCs. (C) RT-qPCR of endogenous *NANOG, OCT4* and *SOX2* expression during OSKM-mediated reprogramming of H9-OCTpGFPdF (OSKM) or H9-OCTpGFPdF by expression of *circBIRC6* (OSKMB) or *circCORO1C* (OSKMC) at the indicated time points post Sendai virus infection. Mean ± SD were determined from quantitative data obtained from three independent experiments.

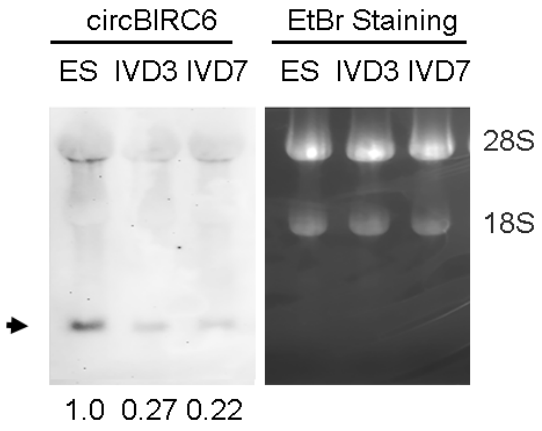

Supplementary Figure 6: Enriched expression of circBIRC6 in undifferentiated hESCs. Northern blotting analysis shows the hybridization of probes to the circular junction of *circBIRC6*. Total RNA was probed from embryoid body-differentiated hESCs at the indicated times. IVD: *in vitro* differentiation day. Arrow indicates the hybridization signal of the RNA probes. EtBr staining of 28S (5070 nt) and 18S(1869nt) rRNA are used as size standards. Quantification of RNA level is shown below each blot as the ratio of signal intensities compared to H9-ES.

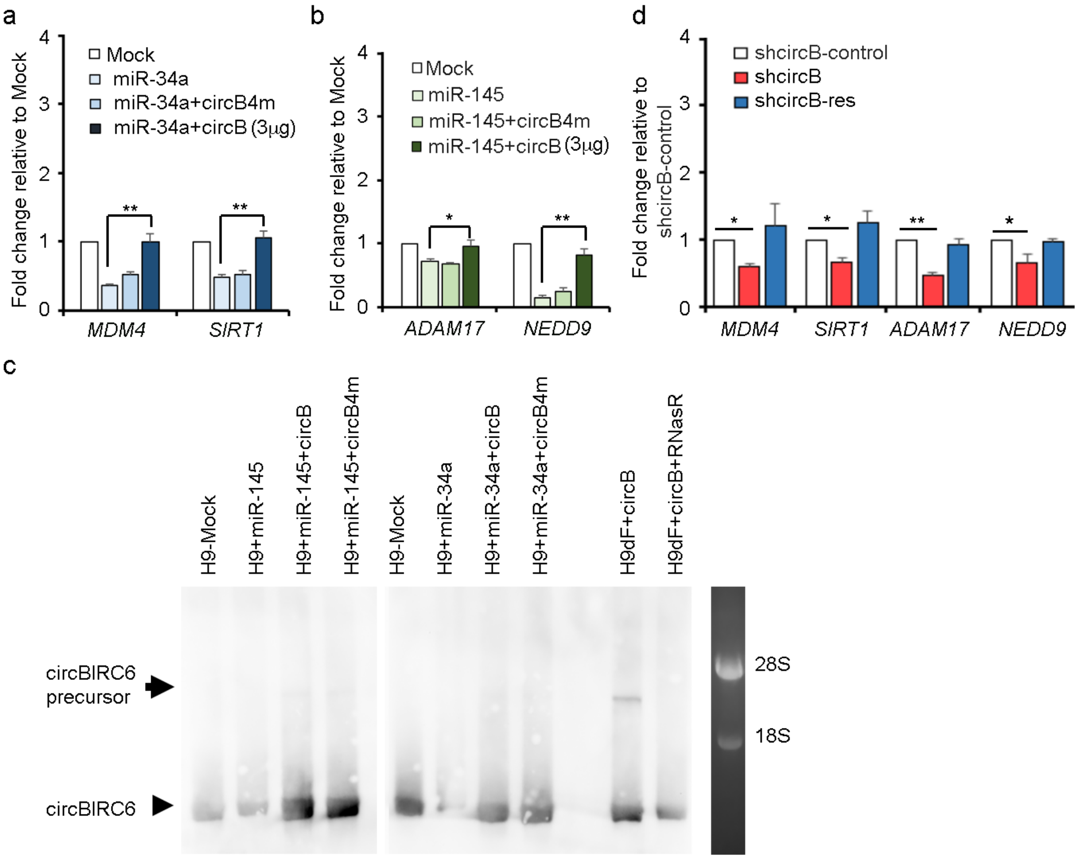

Supplementary Figure 7: Ectopic expression of *circBIRC6* rescued the expression of miR-34a and miR-145 targeting genes. (a) RT-qPCR was used to measure expression of miR-34a target genes (*MDM4* and *SIRT1*) in hESCs transfected with miR-34a mimics, or co-transfected with *circBIRC6* minigene construct [miR-34a+circB (3μg)] or *circBIRC6* minigenes harboring deleted miR-34a and miR-145 binding sites (circB4m) (b) RT-qPCR measurements of expression of miR-145 target genes (*ADMA17* and *NEDD9*) in hESC transfected with miR-145 mimics, or co-transfected with *circBIRC6* minigene construct [miR-145+circB (3μg)] or co-transfected with circB4m. (c) Northern blotting analysis shows probes recognizing *circBIRC6* and its linear precursors hybridized to total RNA isolated from H9 transfected with miRNA mimics and co-transfected with circBIRC6 minigenes (circB) or circBIRC6 minigenes harboring deleted miR-34a and miR-145 binding sites (circB4m). As a control to confirm the efficiency of the probes, detection of circBIRC6 and its linear precursors in total RNA isolated from hESC-derived fibroblasts (H9dF) transfected with circB (H9dF+circB) and RNaseR treated H9dF+circB (H9dF+circB+RNaseR) was performed. EtBr stained rRNA, 28S (5070 nt) and 18S

(1869nt), were used for size standards. (d) RT-qPCR for expression of indicated genes in *circBIRC6* knockdown hESCs (shcircB) or shcircB rescued by *circBIRC6* expression (shcircB-res). Mean ± SD were determined from quantitative data obtained from three independent experiments (*$P < 0.05$, **$P < 0.01$, and ***$P < 0.001$; two-tailed two-sample *t*-test).

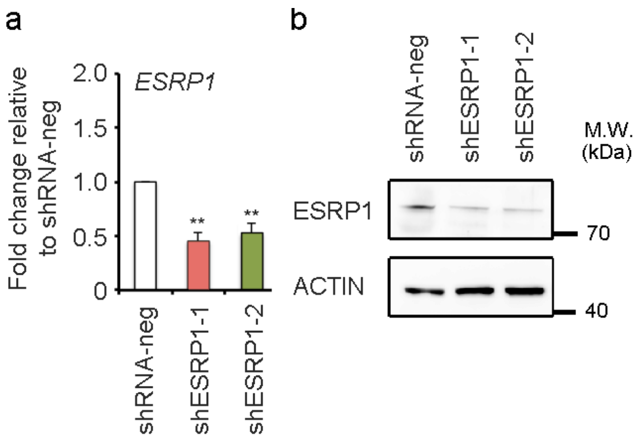

Supplementary Figure 8: Disrupting ESRP1 expression in hESC (H9) by shESRP1s. (a) RT-qPCR and (b) Western blot to measure ESRP1 expression in H9 hESCs transfected with shESRP1s (shESRP1-1 and shESRP1-2). Mean ± SD were determined from quantitative data obtained from three independent experiments (*$P < 0.05$, **$P < 0.01$, and ***$P < 0.001$; two-tailed two-sample *t*-test).

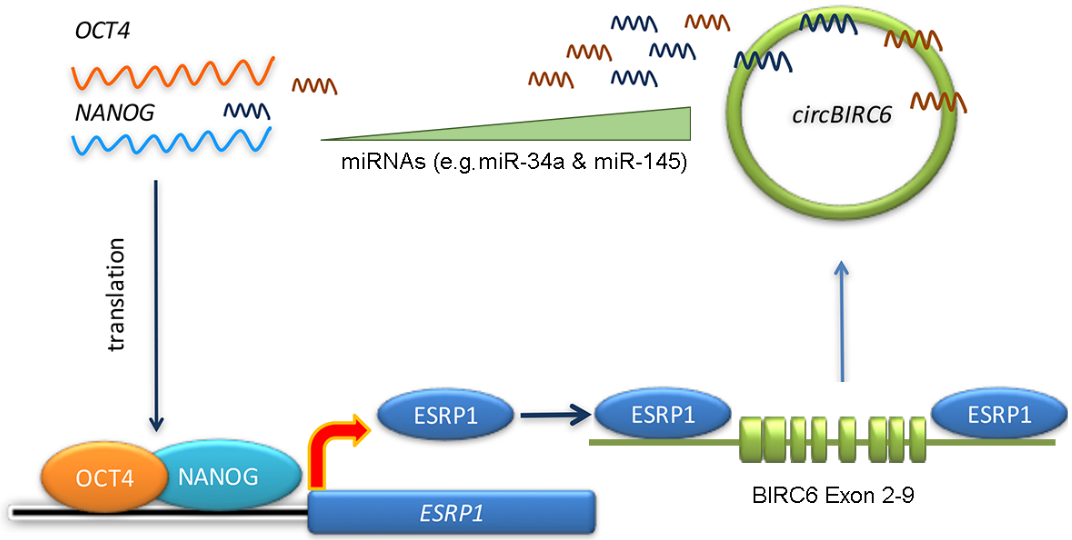

Supplementary Figure 9: Proposed model of the regulatory circuitry in hESCs whereby interactions between the pluripotency associated-transcription factors OCT4 and NANOG, and the splicing factor ESRP1, are coordinated to regulate the expression of a distinct group of circRNAs, including *circBIRC6*, which serve as miRNA sponges to modulate hESC pluripotency status.

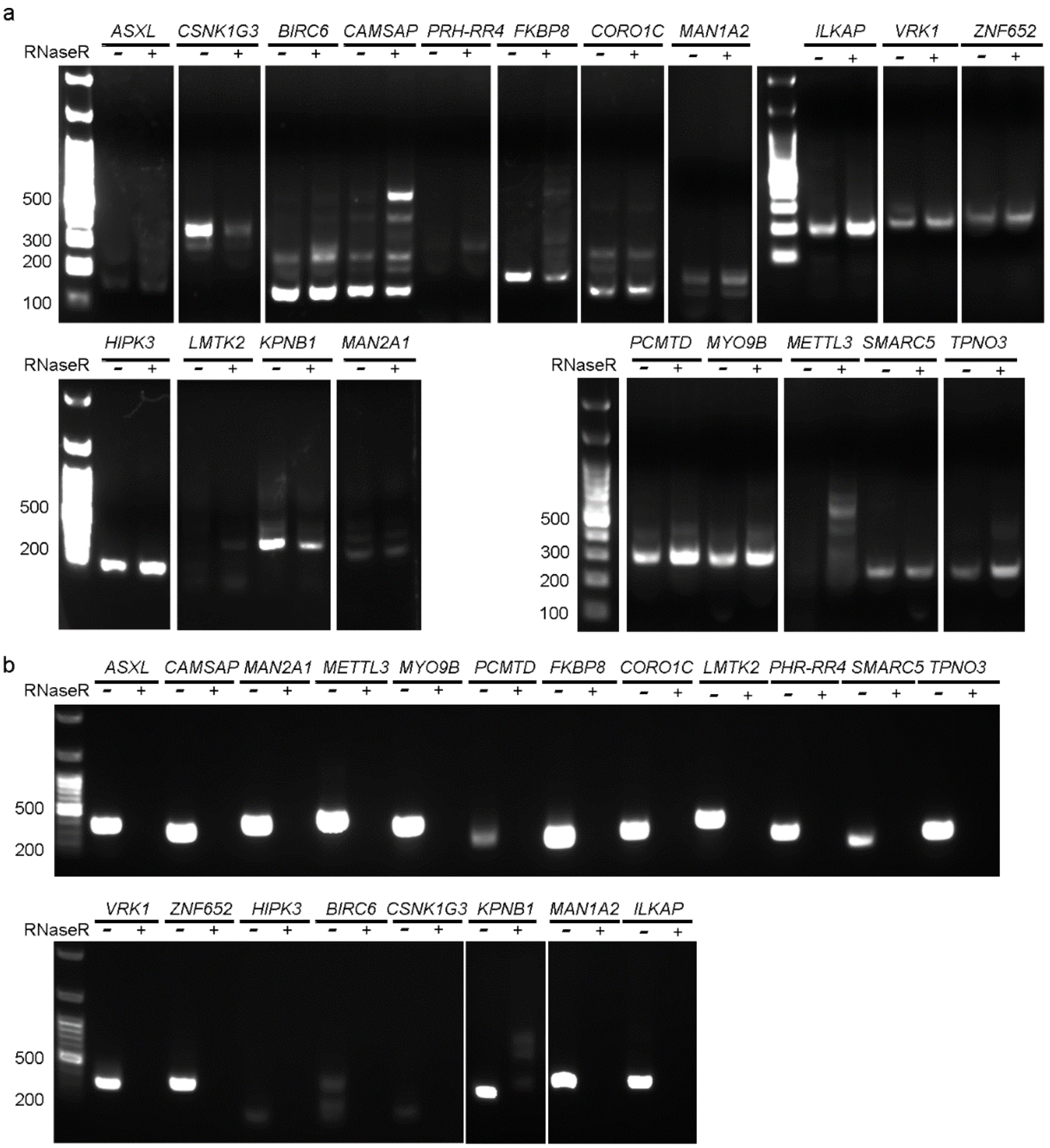

Supplementary Figure 10: RT-PCR of validated circRNAs and their linear counterparts shown in Figure 1. (a) The circular nature of circRNAs was validated with RNaseR treatment (b) The linear counterparts of validated circRNAs were also probed with or without RNaseR treatment.

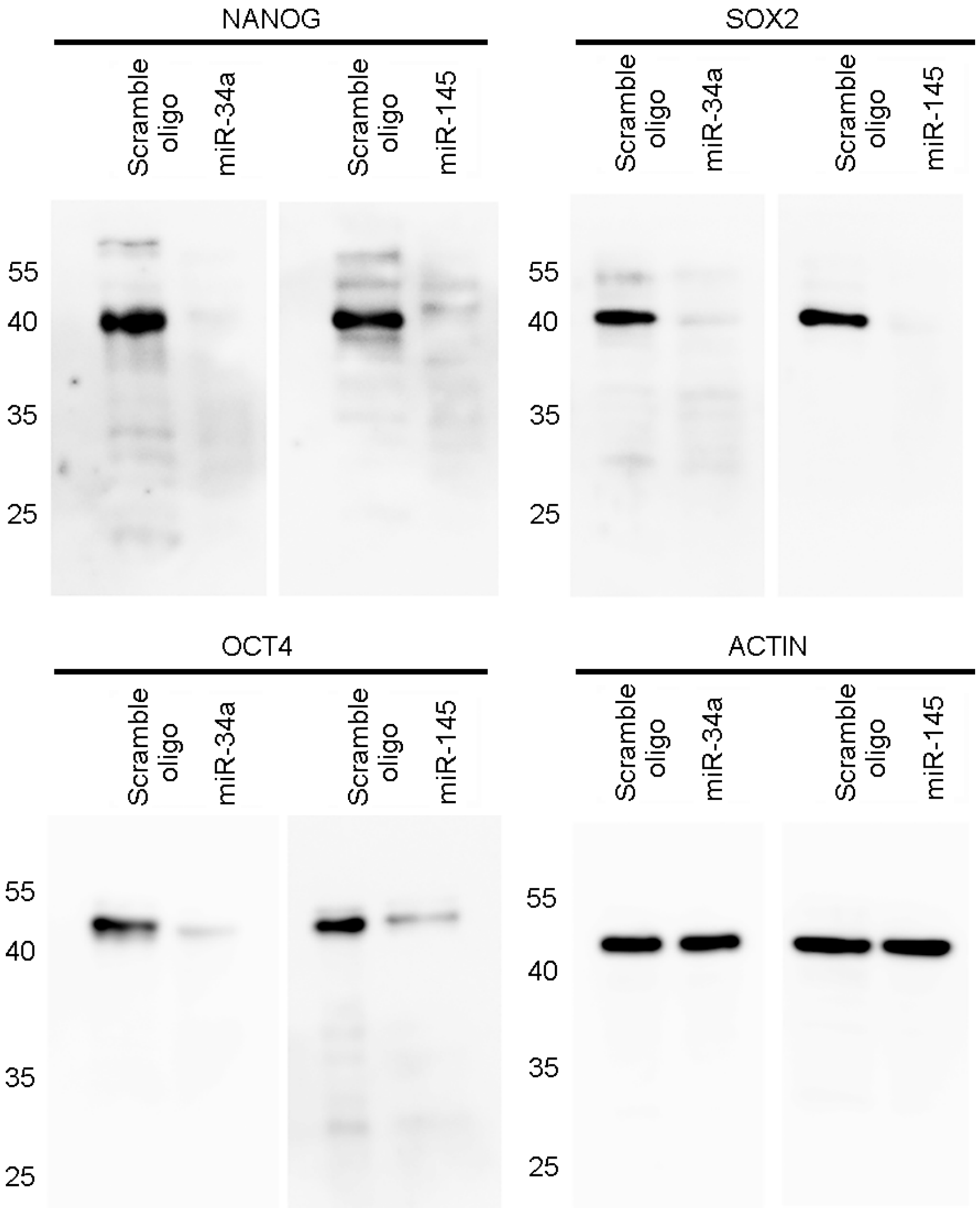

Supplementary Figure 11: Full scans of western blots shown in Figure 5

Supplementary Table 1: Primers used in study

Name	Sequence	Application
cicrASXL1-F	CTCGCATGCCTCAATGCTAT	CircRNA validation
cicrASXL1-R	TGCCTCTATGACCTGCAGAA	CircRNA validation
circBIRC6-F	TCAAGGAGACCAACTTTGGC	CircRNA validation
circBIRC6-R	CTGGAGTTTGCAGAGCAGTG	CircRNA validation
circCAMSAP1-F	CAACGTAGAAAGGGTCTCTGAG	CircRNA validation
circCAMSAP-R	ATGATCAGCATCGAGAAGGTG	CircRNA validation
circCORO1C-F	CCAAGAGGGGACTTGATGTT	CircRNA validation
circCORO1C-R	TGAAGAGCAATTGGTTCCTG	CircRNA validation
circCSNK1G3-F	TGTTTGACCTCCGGATAAGC	CircRNA validation
circCSNK1G3-R	ACCACTAGGTCGTGCCATTC	CircRNA validation
circMAN1A2-F	TCCTAGATGGGCAAAGATGG	CircRNA validation
circMAN1A2-R	TCCAAGGCCTTCTCATGATC	CircRNA validation
circMAN2A1-F	TGAAAGAAGACTCACGGAGGA	CircRNA validation
circMAN2A1-R	TGCTTTGTGAACTTTTCGGA	CircRNA validation
cicrMYO9B-F	GGACCATCCTGGGTGCTG	CircRNA validation
cicrMYO9B-R	TGCCTCTTTCACACTCATCCT	CircRNA validation
circPCMTD1-F	CACTTGTCAGCACCTTGCAT	CircRNA validation
circPCMTD1-R	GGCTTGCTCCACTCTTTCAG	CircRNA validation
circFKBP8-F	TGGTCACTGCTGACTCCAAG	CircRNA validation
circFKBP8-R	TCACCCAGAGTGAACACCAG	CircRNA validation
cicrHIPK3-F	TCGGCCAGTCATGTATCAAA	CircRNA validation
cicrHIPK3-R	TGCTTGGCTCTACTTTGAGTTTC	CircRNA validation
circILKAP-F	GGATACAGAAGGCTGGAGGA	CircRNA validation
circILKAP-R	AGCAAATTTTGAGGCTCGAA	CircRNA validation
circKPNB1-F	CGACTGCAACAGGTTCTTCA	CircRNA validation
circKPNB1-R	GTTCTGCTGCCTCTGAAGCT	CircRNA validation
circPRHRR4-F	TAGCTTGTCTTGCTGGGTCC	CircRNA validation
circPRHRR4-R	CATGTGACAAGAAGGTGCCA	CircRNA validation
circSMARCA5-F	TGGGCGAAAGTTCACTTAGAA	CircRNA validation
circSMARCA5-R	TCTTTGCACCTCTTTCCAAAA	CircRNA validation
circMETTL3-F	GAACTGCTGAAGCTGTGCTG	CircRNA validation
circMETTL3-R	GAGCCAGCCAAGAAATCAAG	CircRNA validation
circLMTK2-F	GCAGTCACGTTTTTCGATGA	CircRNA validation
circLMTK2-R	ATCAGAGTAGGGCTGCTCCA	CircRNA validation
circTNPO3-F	CCCGTAAAGAGGCATGAGAG	CircRNA validation
circTNPO3-R	CGTTCCTTACGAATTGGAGC	CircRNA validation
circVRK-F	AAGGATGATGGCAAATTGGA	CircRNA validation
circVRK-R	TTGGCCAATGGGTAATCCTA	CircRNA validation
circZNF652-F	TTACCAACCATGTGTTTCCG	CircRNA validation
circZNF652-R	TGGGCACAAACAGTTCATGT	CircRNA validation
ASXL1-6292F	CAAACGCATCTTGCTTTTCA	linear isoform
ASXL1-6623R	AGGCAAGGGAAAACACACAC	linear isoform
CAMSAP1-5710F	AGCATGTTTGTCCCAAAAGG	linear isoform

CAMSAP1-5981R	GCTCCACAGAAGCATCAACA	linear isoform
MAN2A1-6298F	GAAGCAAATCCCAGGAATGA	linear isoform
MAN2A1-6640R	CCAAAGCAAAGCATCCTCTC	linear isoform
METTL3-999F	GAGTGCATGAAAGCCAGTGA	linear isoform
METTL3-1399R	CTGCGCATCTCATCATCTGT	linear isoform
MYO9B-7129F	CCCTCCATGAGAGTTTTGGA	linear isoform
MYO9B-7479R	CAGGTTGTCCCTGTTGACCT	linear isoform
PCMTD1-3278F	TTGAATTCCTGGTGACCACA	linear isoform
PCMTD1-3478R	TGCCATGGTCAAAGAATGAA	linear isoform
FKBP8-362F	GGCTGTTGAGGAAGAAGACG	linear isoform
FKBP8-591R	CTTGGAGTCAGCAGTGACCA	linear isoform
ILKAP-477F	GTGAGAGGGAGGAGATGCAG	linear isoform
ILKAP-759R	TTCTGGCTGGAAGCTTGTTT	linear isoform
LMTK2-3533F	TGGTCACAGAGGCACAGAAG	linear isoform
LMTK2-3917R	TTCTGCATTCAGCACACTCC	linear isoform
PRH-480F	ACCATCGAGCTGGAGAAGAA	linear isoform
PRH-750R	AGGGCGAACATTGAGAGCTA	linear isoform
SMARCA5-2705F	TCAGGGAAGCTCTTCGTGTT	linear isoform
SMARCA5-2907R	TTCTTTTTGTGCCTGTGCTG	linear isoform
TNPO3-2999F	CCGACTTTTTGTCGATGGTT	linear isoform
TNPO3-3278R	ACGGAGGTTTCTGATTGTGG	linear isoform
VRK1-1193F	CGGAATGGTCAAACACACAG	linear isoform
VRK1-1506R	CACACGGCTTTGGGATAACT	linear isoform
ZNF652-936F	AGTGAGGAAGAGGCCACAGA	linear isoform
ZNF652-1280R	CTGCATGCGCCTATGAGTAA	linear isoform
linBIRC6-Ex9F	ATCAGGAGACCCAAGCTCAG	linear isoform
linBIRC6-Ex10R	ACTTCCATGGCTTCCTTCTG	linear isoform
linBIRC6-Ex54-F	CAGGCCACAAATTCCGTACT	linear isoform
linBIRC6-Ex55-R	TCTTGAAATCCATTTTCCGC	linear isoform
linBIRC6-Ex1F	GACTTCACTTCCGGCTAACG	linear isoform
linBIRC6-Ex2R	CTGGAGTTTGCAGAGCAGTG	linear isoform
linCORO1C-F	CCAAGAGGGGACTTGATGTT	linear isoform
linCORO1C-R	GGTCAGACTTCCTGGGAACA	linear isoform
linMAN1A2-F	TCCTAGATGGGCAAAGATGG	linear isoform
linMAN1A2-R	CAGCCAATTGCACTGCTTTA	linear isoform
HIPK3-2675F	CTGAGAGTGTGGCTGGTTCA	linear isoform
HIPK3-2816R	ATCCCCACACTAACTGGCTG	linear isoform
CSNK1G3-804F	ACTGGGTCTTCATCGTCTGG	linear isoform
CSNK1G3-1171R	TCCATGCGAGAAATCAGTTG	linear isoform
BIRC6F	TCAAGGAGACCAACTTTGGC	Northern probes
T7-circBIRC6R	TAATACGACTCACTATAGGGCT GGAGTTTGCAGAGCAGTG	Northern probes
T7-preBIRC6R	TAATACGACTCACTATAGGGAC CAAGCCTGGCCTAATTCT	Northern probes
CORO1CF	CCAAGAGGGGACTTGATGTT	Northern probes

T7-circCORO1CR	TAATACGACTCACTATAGGGTG AAGAGCAATTGGTTCCTG	Northern probes
T7-preCORO1CR	TAATACGACTCACTATAGGGAG GCAGGAGAATTGCTTGAA	Northern probes
MAN1A2F	TCCTAGATGGGCAAAGATGG	Northern probes
T7-circMAN1A2R	TAATACGACTCACTATAGGGTC CAAGGCCTTCTCATGATC	Northern probes
T7-preMAN1A2R	TAATACGACTCACTATAGGGAA TGGCAAAATCCTGGAGAA	Northern probes
Guide RNA1	GCTGGAGCAAACCCGGAGG	H9-OCT4pGFP
Guide RNA2	CTTCACGGCACCAGGGGTGA	H9-OCT4pGFP
shcircBIRC6	TCAGATTCTGTGACAGCTAAA	shRNA target site
shcircCORO1C	CAAATGTGAGATTGCCAGAAA	shRNA target site
shMAN1A2	GATTTCAGTGTGGGAAGAGGA	shRNA target site
shcircBIRC6-control	TCAGATTCTGTGACAGGATTT	shcircRNA control
shcircCORO1C-control	CAAATGTGAGATTGCCACTTA	shcircRNA control
shcircMAN1A2-control	GATTTCAGTGTGGGAACTCCA	shcircRNA control
shcircB-2	ACAGATTGTCTTACCTCTTAC	shRNA target site
shLinB	GCTCATTTGTTGGTTTCAGAT	shRNA target site
shESRP1-1	CCGGTATATTGAGGTTTACAA	shRNA target site
shESRP1-2	GCATAAAGACTTGTTGGGTAA	shRNA target site
shRNA-neg	CCTAAGGTTAAGTCGCCCTCG	shRNA target site
preBIRC6-F	TCCAGTTGGCCCAATAATGT	RT-qPCR
preBIRC6-R	CTGGAGTTTGCAGAGCAGTG	RT-qPCR
preCORO1C-F	ATCCCTGTTGCTGTTTCCAT	RT-qPCR
preCORO1C-R	TGAAGAGCAATTGGTTCCTG	RT-qPCR
preMAN1A2-F	CTTAGGCATCCAGGGTTCCT	RT-qPCR
preMAN1A2-R	TCCAAGGCCTTCTCATGATC	RT-qPCR
CDR1as	CCCAGTCTTCCATCAACTGG	RT-qPCR
CDR1as	AGACATGGATTGTCCGGAAG	RT-qPCR
ESRP1-F	TCGTACTCATGGGGTTCACA	RT-qPCR
ESRP1-R	AATGCTCTGTCCGCAGACTT	RT-qPCR
HNRNPA1-F	ACGAAACCAAGGTGGCTATG	RT-qPCR
HNRNPA1-R	GTGCTTGGCTGAGTTCACAA	RT-qPCR
SNRNP1-F	CAGGGAAGAGGCACTGTAGC	RT-qPCR
SNRNP1-R	GGGTGGTCTCATACCAGGTG	RT-qPCR
ESRP1-A-F	TACTCATAACACTTGAACTC	RIP
ESRP1-A-R	AGTATTAAATCATAACTAAA	RIP
ESRP1-C-F	GGCAAAATAATATACTTCG	RIP
ESRP1-C-R	TGCTATATATCAGGAAATGC	RIP
ESRP1-B-F	AACCAGGTGGACAGGTGAAA	RIP
ESRP1-B-R	CTGGAGTTTGCAGAGCAGTG	RIP
NANOG-F	CAGCAGATGCAAGAACTCTCCA	RT-qPCR

NANOG-R	CATTGCTATTCTTCGGCCAGT	RT-qPCR
OCT4-F	CCGAAAGAGAAAGCGAACCAG	RT-qPCR
OCT4-R	AGAACCACACTCGGACCACATC	RT-qPCR
SOX2-F	CGATGCCGACAAGAAAACTT	RT-qPCR
SOX2-R	CAAACTTCCTGCAAAGCTCC	RT-qPCR
KLF4-F	GCGCTGCTCCCATCTTTCTC	RT-qPCR
KLF4-R	GGGGAAGTCGCTTCATGTGG	RT-qPCR
MYC-F	CCTGGTGCTCCATGAGGAGA	RT-qPCR
MYC-R	GAGCCTGCCTCTTTTCCACAG	RT-qPCR
GSC-F	CGGAGAAGTGGAACAAGACGT	RT-qPCR
GSC-R	GCGTGTGCAAGAAAGTAGCATC	RT-qPCR
MIXL1-F	AACGAAATGTCTGAAGCCCCA	RT-qPCR
MIXL1-R	TCCTCCCATGAGTCCAGCTTT	RT-qPCR
Brachyury-F	CCAATGAGATGATCGTGACCA	RT-qPCR
Brachyury-R	ATTCCCCGTTCACGTACTTCC	RT-qPCR
SOX17-F	TTCGTGTGCAAGCCTGAGAT	RT-qPCR
SOX17-R	GTGTGTAACACTGCTTCTGGCC	RT-qPCR
PAX6-F	AACAGACACAGCCCTCACAAAC	RT-qPCR
PAX6-R	CGGGAACTTGAACTGGAACTGA	RT-qPCR
SOX1-F	TGGCATCTAGGTCTTGGCTCA	RT-qPCR
SOX1-R	GCACGAAGCACCTGCAATAAG	RT-qPCR
MDM4-F	GCACCCAGCCAAGTTGTAAT	RT-qPCR
MDM4-R	GAGGATTCTTTCATGGGCAA	RT-qPCR
SIRT1-F	AGCTGGATTTGGGACTGATG	RT-qPCR
SIRT1-R	GCTGGTGGAACAATTCCTGT	RT-qPCR
NEDD9-F	ATGTCCACGTCTTCCACCTC	RT-qPCR
NEDD9-R	TTAGGCTGGAGACACCCATC	RT-qPCR
ADAM17-F	ACAGCCATGGAGGTGTTTGT	RT-qPCR
ADAM17-R	ATCCGGATCATGTTCTGCTC	RT-qPCR
ESRP1-P(-2)F	AACTAGCAGCCAGGACCAAA	RT-qPCR
ESRP1-P(-2)R	CACCTAGTCACTCCCCAGGA	RT-qPCR
ESRP1-P(-1)F	TCCTGGGGAGTGACTAGGTG	RT-qPCR
ESRP1-P(-1)R	CAGTGCAAACAGCAGGAGAA	RT-qPCR
ESRP1-P(0)F	AAAGCCACATCCCAAAACAG	RT-qPCR
ESRP1-P(0)R	CTATGCAAAAAGCCTGGAGC	RT-qPCR
ESRP1-P(1)F	CGGATTACTTGGTGGTGCTT	RT-qPCR
ESRP1-P(1)R	CACGACTTTCCAGAACAGCA	RT-qPCR
ESRP1-P(2)F	TGACCCGTGCTTCTCTACCT	RT-qPCR
ESRP1-P(2)R	TCTTCTTTGCAGTCCTCCGT	RT-qPCR
circEGFREx25-F	AGGACTTGGCAAATGCATTC	RT-PCR
circEGFREx25-R	CCACCACGTCGTCCATGTCT	RT-PCR
pre-EGFREx25-F	TAGCATCTCTACGGGCCAT	RT-PCR
circZEBEx11-F	GATCAACCACCAATGGTTCC	RT-PCR
circZEBEx11-R	TTGCAGTTTGGGCATTCATA	RT-PCR
pre-ZEBEx11-F	TGCTGAGAAGATTCCATGATTG	RT-PCR
