# Supplementary material for: The circular RNA circBIRC6 participates in the molecular circuitry controlling human pluripotency
Source: Nat Commun. 2017 Oct 27;8:1149. doi: 10.1038/s41467-017-01216-w (PMC5658440; doi:10.1038/s41467-017-01216-w)
Supplement: Supplementary file 2 — Description of Additional Supplementary Files [file 41467_2017_1216_MOESM2_ESM.pdf]

Description of Supplementary Files

File name: Supplementary Data 1
Description: 61 cricRNA candidates with highest reads supporting circular junction.

File name: Supplementary Data 2
Description: The Probability of Interaction by Target Accessibility (PITA) algorithm predictes miRNA binding sites on circBIRC6

File name: Supplementary Data 3
Description: The microarray analysis of splicing factors expression in hESC H9, hiPSC CFB50 and it parential cells (HF). (Fold change>5)
